# Representing connectivity: quantifying effective habitat availability based on area and connectivity for conservation status assessment and recovery

**DOI:** 10.7717/peerj.622

**Published:** 2014-10-09

**Authors:** Maile Neel, Hayley R. Tumas, Brittany W. Marsden

**Affiliations:** 1 Department of Plant Science and Landscape Architecture, University of Maryland, USA; 2 Department of Entomology, University of Maryland, USA; 3 Warnell School of Forestry and Natural Resources, University of Georgia, Athens, GA, USA; 4 Marine Estuarine and Environmental Science Graduate Program, University of Maryland, College Park, MD, USA

**Keywords:** Endangered species act, Reserve design, Graph theory, Connectivity, Fragmentation

## Abstract

We apply a comprehensive suite of graph theoretic metrics to illustrate how landscape connectivity can be effectively incorporated into conservation status assessments and in setting conservation objectives. These metrics allow conservation practitioners to evaluate and quantify connectivity in terms of representation, resiliency, and redundancy and the approach can be applied in spite of incomplete knowledge of species-specific biology and dispersal processes. We demonstrate utility of the graph metrics by evaluating changes in distribution and connectivity that would result from implementing two conservation plans for three endangered plant species (*Erigeron parishii*, *Acanthoscyphus parishii* var. *goodmaniana*, and *Eriogonum ovalifolium* var. *vineum*) relative to connectivity under current conditions. Although distributions of the species differ from one another in terms of extent and specific location of occupied patches within the study landscape, the spatial scale of potential connectivity in existing networks were strikingly similar for *Erigeron* and *Eriogonum*, but differed for *Acanthoscyphus*. Specifically, patches of the first two species were more regularly distributed whereas subsets of patches of *Acanthoscyphus* were clustered into more isolated components. Reserves based on US Fish and Wildlife Service critical habitat designation would not greatly contribute to maintain connectivity; they include 83–91% of the extant occurrences and >92% of the aerial extent of each species. Effective connectivity remains within 10% of that in the whole network for all species. A Forest Service habitat management strategy excluded up to 40% of the occupied habitat of each species resulting in both range reductions and loss of occurrences from the central portions of each species’ distribution. Overall effective network connectivity was reduced to 62–74% of the full networks. The distance at which each CHMS network first became fully connected was reduced relative to the full network in *Erigeron* and *Acanthoscyphus* due to exclusion of peripheral patches, but was slightly increased for *Eriogonum*. Distances at which networks were sensitive to loss of connectivity due to presence non-redundant connections were affected mostly for *Acanthoscyphos*. Of most concern was that the range of distances at which lack of redundancy yielded high risk was much greater than in the full network. Through this in-depth example evaluating connectivity using a comprehensive suite of developed graph theoretic metrics, we establish an approach as well as provide sample interpretations of subtle variations in connectivity that conservation managers can incorporate into planning.

## Introduction

Because habitat destruction and degradation are leading causes of species decline and extinction (e.g., Wilcove et al., 1998), conservation strategies often include establishing networks of protected areas to slow rates of habitat loss and fragmentation (Margules & Pressey, 2000; Simberloff, 1998; Soulé & Simberloff, 1986). Key features of such networks for individual species include *representation* of *resilient* populations with sufficient *redundancy* to facilitate species persistence (referred to as the ‘Rs’; Shaffer & Stein, 2000). Larger total amounts of habitat as well as larger habitat patches are considered more resilient due to the higher probability of supporting large (Jagers & Harding, 2009), genetically diverse (Frankham, 2005) populations that are more able to withstand perturbations through tolerance, acclimation, or adaptation (Bengtsson et al., 2003; Holling, 1973). Representing the ecological and geographic ranges of a species captures diversity for future adaptation and lowers risk of similar conditions affecting all populations simultaneously (reviewed in Noss, O’Connell & Murphy, 1997). Redundancy from representing multiple resilient sites spreads risk of population loss from stochastic events and can decrease chances of synchronous dynamics occurring at all sites throughout a species range (Stacey & Taper, 1992). Maintaining ecological connectivity among sites contributes to their resiliency by allowing continuance of ecological and evolutionary processes that relate to dispersal. These processes facilitate persistence over short timeframes through demographic rescue or recolonization after local decline or extirpation, and over longer timeframes through benefits of ongoing gene flow (Hanski & Simberloff, 1996). The contrasting conditions (declining area of occupancy and increasing fragmentation) are thus of concern because they increase extinction risk. As such, they are factors in ranking species endangerment under the IUCN Red List criteria (IUCN, 2010; Mace et al., 2008) and assessing biodiversity status under the Convention on Biological Diversity (Butchart et al., 2010). The 3 Rs have been adopted as guiding principles for recovery of species listed under the US Endangered Species Act (ESA; National Marine Fisheries Service, 2010). Despite their obvious benefits, means of converting the 3 Rs from philosophical guidelines into comprehensive, objective and measurable criteria that are required under the ESA have been wanting. Changes in representation and redundancy of habitat area and extent, based on sites that remain or would be conserved compared to a chosen baseline are relatively easy to quantify objectively (e.g., numbers of hectares occupied, numbers of sites occupied, or size of range can be counted). However, adequately quantifying connectivity (Fahrig, 2002) and determining how it would change under different landscape patterns is less than straightforward. Here we demonstrate a graph theoretic approach based on habitat availability and configuration that can be used by conservation practitioners to develop criteria related to connectivity from the 3 R principles that evaluate how well a network of sites represents a baseline.

Habitat distribution has been increasingly used for conservation planning and status assessment because of the fundamental importance of habitat area and connectivity for species persistence and because species distribution data are often more available than are data on individual abundance and population trajectories (Attorre et al., 2013; Noss, O’Connell & Murphy, 1997). Despite development of many metrics for quantifying landscape pattern (e.g., McGarigal et al., 2002; Neel, McGarigal & Cushman, 2004), practical holistic definitions of fragmentation and means of objectively quantifying the effects of loss or gain of habitat patches on connectivity have remained elusive. Difficulty arises in part from the complexity of separating the joint and independent effects of area and isolation (Fahrig, 1997). Consequently, definitions in species assessments are mostly qualitative and generic. For example, the IUCN (2010) defines a species as “severely fragmented” if >50% of its occurrences are isolated at a scale appropriate to the species. No distinction is made between natural and anthropogenic isolation or assessment of changes in isolation. We know qualitatively that most species listed under the US ESA have been reduced in range extent, and habitat amount and connectivity; but magnitude of losses have most often not been quantified (Leidner & Neel, 2011; Neel et al., 2012). Habitat-based conservation goals for many species are similarly vague and commonly include maintaining “natural” or current levels of connectivity or reflecting historical connectivity (Rubio & Saura, 2012). Recovery plans for US endangered species often include qualitative goals of maintaining distributions and potential for dispersal, but few have recovery criteria specifically related to fragmentation. For example, recovery criteria for 36 of the 1,173 species that have recovery plans as of 2010 include explicit qualitative statements regarding maintaining connectivity; 30 species have qualitative statements about habitat amount. Only 7% of species with plans had quantitative criteria for habitat amount (Neel et al., 2012). Only six species in one multispecies plan for plants endemic to gabbro soils had quantitative criteria for fragmentation (M Neel & A Leidner, 2012, specifically, fragmentation may not increase by more than 5%; unpublished data). This recovery plan, however, did not specify how changes in connectivity would be assessed (US Fish & Wildlife Service, 2002a). We show here how this graph theoretic approach to evaluating species status and setting conservation criteria based on connectivity and habitat availability overcomes these existing shortcomings.

Graph theory provides an effective means of quantifying connectivity by identifying patches (a.k.a. *nodes*) that would be linked (via *edges*) into *components* at a specified threshold distance (e.g., Calabrese & Fagan, 2004; Urban & Keitt, 2001). All habitat patches within a component are considered available to organisms capable of dispersing that distance. Graph metrics are well supported theoretically (Pascual-Hortal & Saura, 2006) and particular ones have been used to evaluate patch importance (Baranyi et al., 2011; Bodin & Saura, 2010; Saura & Rubio, 2010) and to evaluate the contribution of changes in connectivity versus habitat loss in forest networks (Saura et al., 2011). Unfortunately, most graph metrics have the same non-intuitive behavior common to many landscape pattern metrics that yields ambiguous or inaccurate interpretations. Problems include increasing in value as the number of patches (i.e., amount of fragmentation) increases or spatial scale decreases, having non-monotonic relationships along gradients of area and aggregation, confounding area and isolation, and failing to reflect the full fragmentation gradient by being applicable only in landscapes with multiple patches (Ferrari, Lookingbill & Neel, 2007; Pascual-Hortal & Saura, 2006; Saura & Pascual-Hortal, 2007). Two graph-theoretic metrics—the Integrated Index of Connectivity (*IIC*) and Probability of Connectivity (*PC*)—have overcome these issues (Table 1; Pascual-Hortal & Saura, 2006). These area- (or quality) and distance-weighted measures accurately describe habitat availability or “reachability” as a function of dispersal distance across the entire gradient of habitat area and isolation (Pascual-Hortal & Saura, 2006). Values are highest when a landscape comprises one large habitat patch and decline appropriately as area decreases or is apportioned into separate patches. *IIC* is a binary index that considers all patches within a given threshold distance to be connected, whereas in *PC* there is a user-specified declining probability of connection across increasing distances (Table 1; Pascual-Hortal & Saura, 2006). Derivatives of *IIC* and *PC* allow separation of area and isolation effects and have been used to evaluate patch importance (Baranyi et al., 2011; Bodin & Saura, 2010; Saura & Rubio, 2010).

**Table 1:** Graph theoretic metrics used to quantify network extent and connectivity. Description of the graph theoretic measures that are used to quantify habitat availability as a function of habitat amount and spatial configuration (from Saura & Torné, 2012; Saura & Rubio, 2010) and example conservation criteria that relate the measures to the 3-Rs (representation, redundancy and resilience; Shaffer & Stein, 2000).

Metric	Formula	Meaning	Range of values	Evaluation of difference	Example 3R criteria
*A*	∑*a_i_*	Total area of patches in the landscape	Unbounded sum of area in all patches	Absolute and percent difference	Conserve *x* hectares of high density occupied habitat by preventing mining disturbance
*Number of patches*		Number of discrete patches on landscape	1 to unbounded	Absolute and percent difference	Conserve y% of the patches known compared to a chosen baseline (eg., at listing or historically)
*NC*		Number of components in the landscape	1 to number of patches	Absolute and percent difference	Conserve patches that link components together, given a maximum (or median) dispersal distance of *x* meters
*dA*	$\frac{{A}_{b a s e}-{A}_{c h a n g e d}}{{A}_{b a s e}}$	Proportional change in network area in two landscapes	0–1	Used in conjunction with *dEC(IIC)*	Increase area to *x*% of the area at time of listing
*PC*	$\frac{1}{{A}_{L}^{2}}\ast \sum _{i=1}^{n}\sum _{j=1}^{n}{a}_{i}\times {a}_{j}\times {p}_{i j}^{\ast }$	Probability that two individuals dropped randomly in a landscape will be in the same component (and thus would be able to ‘reach’ each other)	0–1	Not used directly—used to calculate *PC_num_*	N/A
*IIC*	$\frac{1}{{A}_{L}^{2}}\ast \sum _{i=1}^{n}\sum _{j=1}^{n}\frac{{a}_{i}\times {a}_{j}}{1+{n l}_{i j}}$ where *nl_ij_* represents the number of links that must be passed to move from patch *i* to *j*	Index has a value of 1 when the entire landscape is occupied by a single patch	Proportion of the landscape occupied by the largest patch—to1	Not used directly—used to calculate *IIC_num_*	N/A
*IIC_num_*	$\sum _{i=1}^{n}\sum _{j=1}^{n}\frac{{a}_{i}\times {a}_{j}}{1+{n l}_{i j}}$ where *nl_ij_* represents the number of links that must be passed to move from patch *i* to *j*	Habitat available or reachable as a function of being within the specified dispersal distance	Unbounded function of area patches and the links between them	Not used directly—used to calculate the *EC(IIC)* metrics and the *varIIC* metrics	N/A
*EC(IIC)*	$\sqrt{\sum _{i=1}^{n}\sum _{j=1}^{n}\frac{{a}_{i}\ast {a}_{j}}{1+{n l}_{i j}}}$ where *nl_ij_* represents the number of links that must be passed to move from patch *i* to *j*	The area of a single patch of habitat that would yield the same *IIC* value as is seen in the observed landscape	Area of the largest patch to less than total habitat area	Absolute and percent difference	Increase *EC(IIC)*across all distance thresholds from *x* to *y* km by 10% over values at chosen baseline
*dEC(IIC)*	$\frac{{E C(I I C)}_{b a s e}-{\hspace{0.167em} E C\left(I I C\right)}_{c h a n g e d}}{{E C(I I C)}_{b a s e}}$	Proportional change in *EC(IIC)* between two scenarios. Represents the changes in both area and isolation. Interpretation is more straightforward if the landscape with smaller area is subtracted from the larger to yield a positive numerator	0-1	Proportional difference; when used in conjunction with *dA* it allows assessment of the rate of change that is due to area versus isolation	See *EC(IIC)* above
*d(dA-dEC(IIC))*	*dA-dEC(IIC)*	*dA>dEC(IIC)*—changes between networks are due to area of isolated patches *dA<dEC(IIC)*—changes between networks are due to loss/gain in stepping stone patches that are important for connectivity beyond simply their area *dA*≈*dEC(IIC)*—changes between networks are due habitat that is contiguous with other habitat	0-Unbounded	Difference	Conserve habitat patches such that *dA-dEC(IIC)* is minimized
*varIIC_k_*	*IIC_num_*−*IIC*_*num*−*remove*_	Absolute difference between the *IIC_num_* value of the full network and when patch *k* is removed	Unbounded	Absolute and percent difference	Conserve patch *k* to prevent *x*% reduction in *IICnum*
*sum_varIIC_k_*	∑*IIC_num_*−*IIC*_*num*−*remove*_	Sum of *varIIC* values across all patches in a landscape	Unbounded		
*sum_varIIC_intra_*	*a_i_* × *a_j_* when *i* = *j* = *k* where *k* is the removed patch	The sum across all patches of the contribution of patch *k* in terms of intrapatch connectivity; equivalent to area-weighted mean patch size based metrics	Square of total patch area if no patches are connected through links	Absolute and percent difference	Conserve integrity of largest patches to facilitate within patch movement so that *sum_varIIC_intra_* is not reduced by more than *x*%. **OR** Restore patches to increase extent of continuous habitat so that *sum_varIIC_intra_* is increased by at least *x*%
*sum_varIIC_flux_*	$\sum \frac{{a}_{i}\times {a}_{j}}{1+{n l}_{i j}}$ when *i* = *k*, or *j* = *k* and *i* ≠ *j* where *nl_ij_* represents the number of links that must be passed to move from patch *i* to *j* and *k* is the removed patch	The sum across all patches of the area-weighted dispersal flux through the connections of patch *k* to or from all of the other patches in the landscape when *k* is either the starting or ending patch of that connection or flux	Unbounded; a function of patch area and numbers of links between patches	Absolute and percent difference	Maintain redundancy in connections among large patches at key dispersal distances such that *sum_varIIC_flux_* in reserve plans is not reduced more than *x*%
*sum_varIIC_connector_*	$\sum \frac{{a}_{i}\times {a}_{j}}{1+{n l}_{i j}}$ when *i* ≠ *k*, *j* ≠ *k*, and *k* is part of link *nl_ij_* where *nl_ij_* represents the number of links that must be passed to move from patch *i* to *j* and *k* is the removed patch	The sum across all patches of the contribution of patch *k* or link *l* as a connecting element or stepping stone through the best path between two other habitat patches (*i* and *j*). Depends only on topological position—needs to connect at least two other patches (i.e., is part of *nl_ij_*)	Unbounded; a function of patch area and numbers of links between patches	Absolute and percent difference	Restore habitat in locations that create redundancies in connection so that peaks in *sum_varIIC_connector_* are eliminated in distance ranges that are relevant to species dispersal. **OR** Prevent increases in *sum_varIIC_connector_* of >10% in the dispersal range of the species
*theta_varIIC_intra_*	*sum_varIIC_intra_*/*sum_varIIC* × 100	Relative contribution of changes in connectivity due to area within patches when a patch is left of a network	Maximum = 100	Absolute and percent difference from baseline or between landscapes	See criteria for *sum_varIIC_intra_*
*theta_varIIC_flux_*	*sum_varIIC_flux_*/*sum_varIIC* × 100	Relative contribution of changes in connectivity due to changes in *sum_varIIC_flux_* when patches are left of a network	Maximum = 100 − *theta_varIICintra*	Absolute and percent difference from baseline or between landscapes	See criteria for *sum_varIIC_flux_*
*theta_varIIC_connector_*	*sum_varIIC_connector_*/*sum_varIIC* × 100	Relative contribution of changes in connectivity due to changes in *sum_varIIC_connector_* when a patch is left of a network	Maximum = 100 − (*theta_varIIC_intra_* +*theta_varIIC_flux_*)	Absolute and percent difference from baseline or between landscapes	See criteria for *sum_varIIC_connector_*

Here we show how these metrics can be combined into a comprehensive analysis to assess species status and to develop objective and measurable conservation criteria related to connectivity aspects of the 3 R conservation principles. We do so by evaluating the effects of losing habitat excluded from two reserve networks for each of three species listed under the US ESA. Specifically, we (1) quantify multiple aspects of potential network connectivity in current species distributions using habitat availability as measured by a standardized form of *IIC* and additive fractions of *IIC*; (2) quantify the effects of changes to landscape patterns on connectivity resulting from the conservation strategies; and (3) demonstrate how the findings can be used in conservation assessment and planning.

A major strength of this approach is its flexibility. We apply it in a conservation planning context, but it can be used to evaluate any change in landscape area and configuration based on distribution information from at least two time points or scenarios. Thus it can be used to evaluate predicted changes in distribution under climate change or development scenarios. And although we have presented it in static scenarios, it can be applied to dynamic landscapes. It is appropriate for use across full gradients of area and isolation (i.e., it is not restricted to multi-patch landscapes). It can be used when nothing more is known than location and areal extent of populations or habitat, yet additional data (e.g., habitat quality, population size, or population trajectories) can easily be incorporated (e.g., Looy et al., 2013). Distance matrices can be as basic as Euclidean distances between patches or as complex as probabilities derived from models of movement (e.g., Lookingbill et al., 2010), resistance surfaces reflecting matrix permeability, or actual observations. If dispersal capabilities are known, analyses can focus on species-specific threshold distances. In the more common circumstance in which knowledge about dispersal is incomplete, examining a broad range of distances identifies the distance at which structural changes would occur and potential effects can be inferred based on related or ecologically similar species. For example, if habitat loss would alter network connectivity at a scale of 9–12 km, there would be no large effects on species that regularly disperse either 30 km or only 500 m. By contrast, for species with a median dispersal distance of 10 km, habitat loss could convert the landscape from connected to disconnected. Analysis of basic structural connectivity can also be used to determine if connectivity changes at different threshold distances affect other species (e.g., pollinators or seed dispersers) or ecological processes on which a target species depends.

**Figure 1:**
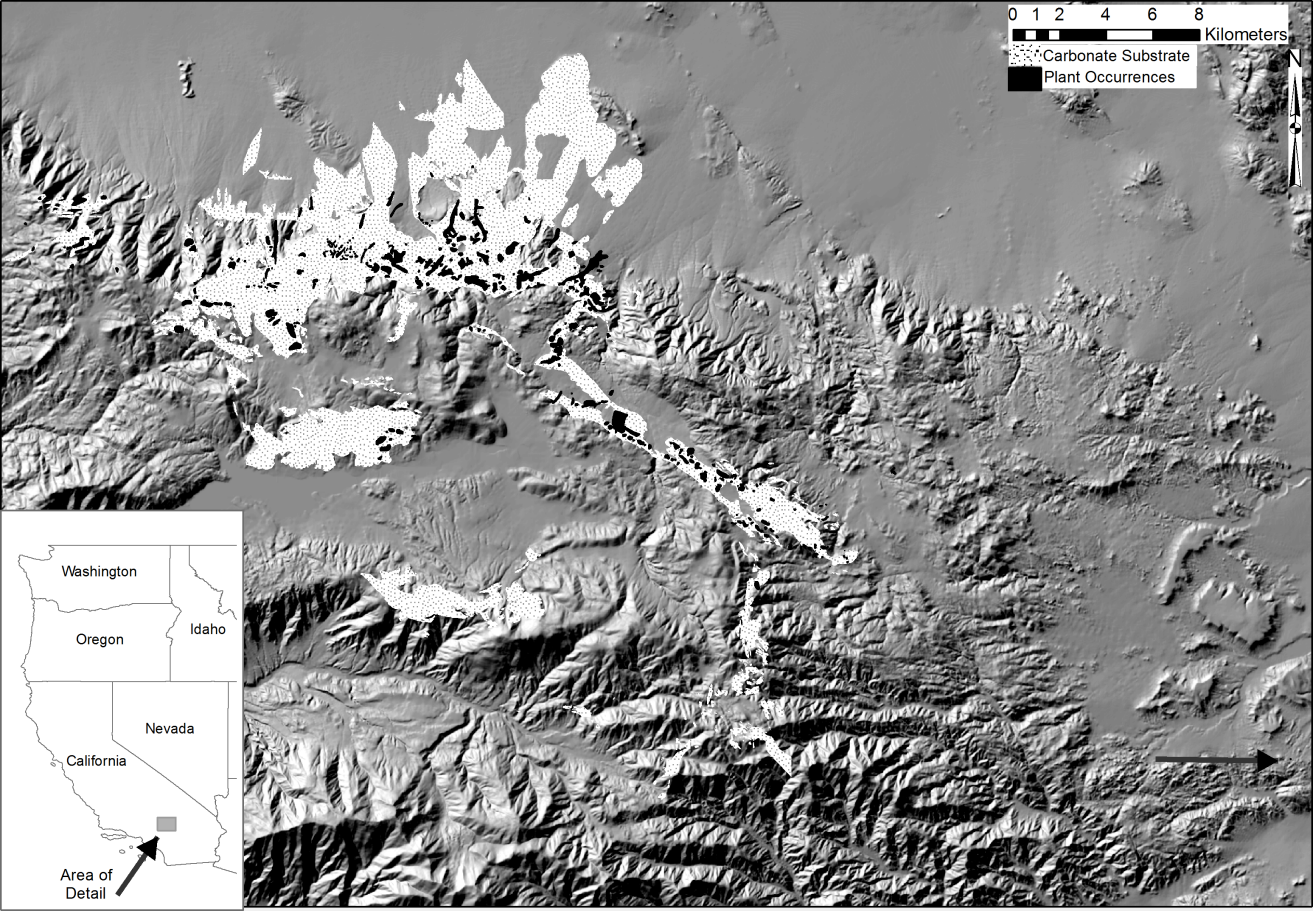
Combined distribution map for *Erigeron parishii*, *Eriogonum ovalifolium* var. *vineum*, and *Acanthoscyphus parishii* var. *goodmaniana*. The three taxa, (indicated by black polygons) occur almost exclusively on carbonate (limestone and dolomite) substrates (stippled areas) in the eastern San Bernardino Mountains of southern California, USA. The arrow in the lower right points out a small patch of *Erigeron parishii* habitat.

**Figure 2:**
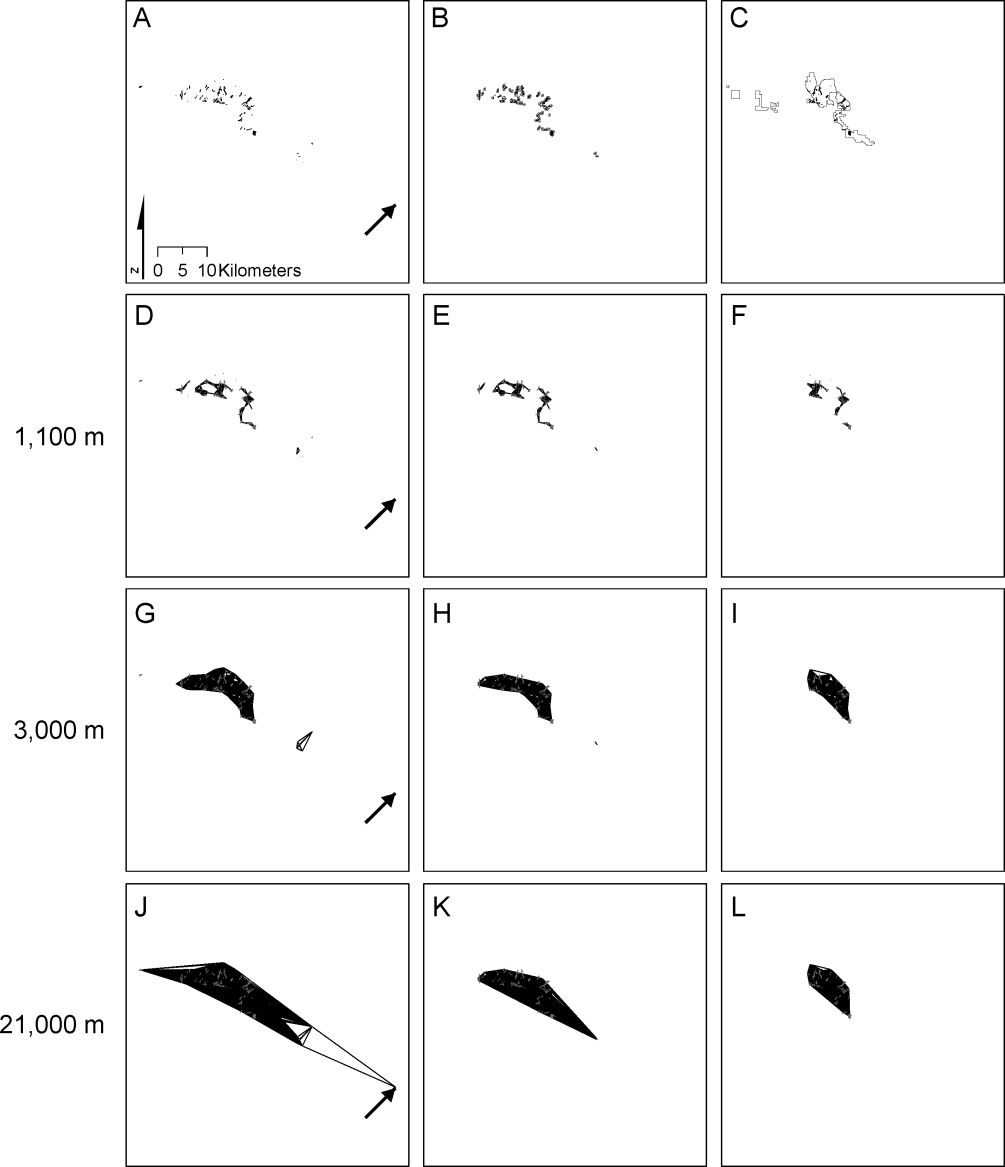
Distribution of and connectivity among *Erigeron parishii* patches. The full species distribution (A) is compared with patches included in critical habitat (B) and the CHMS reserve (C). Edges connecting patches at distances of 1,100 m (D, E, F), 3,000 m (G, H, I), and 21,000 m (J, K, L) for the full distribution (A, D, G, J), critical habitat (B, E, H, K) and CHMS reserve (C, F, I, L) are presented. The arrow in the lower right of (A, D, G, J) points out a small patch of *Erigeron parishii* habitat that is only present in the full species distribution.

**Figure 3:**
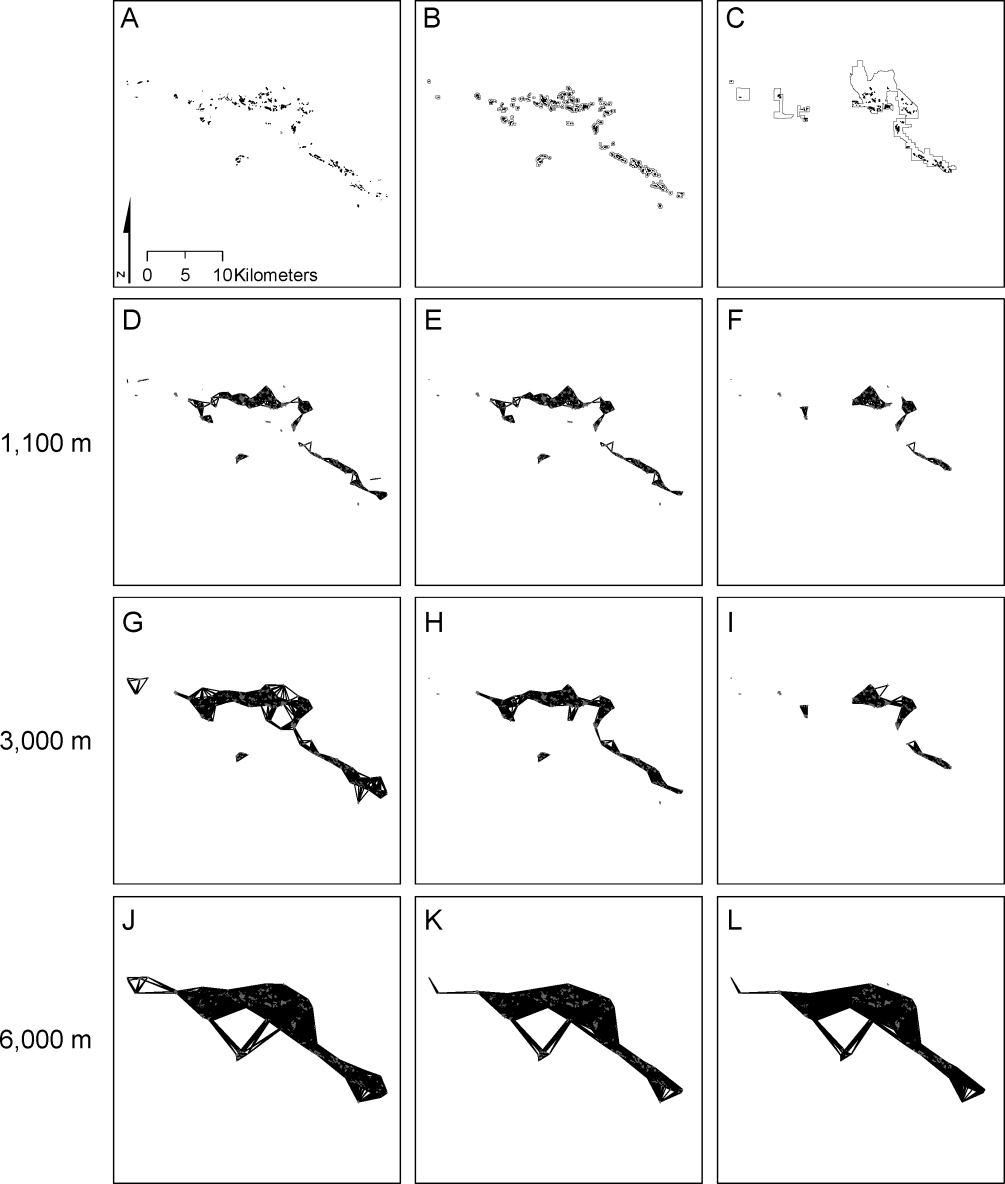
Distribution of and connectivity among *Eriogonum ovalifolium* var. *vineum* patches. The full species distribution (A) is compared with patches included in critical habitat (B) and the CHMS reserve (C). Edges connecting patches at distances of 1,100 m (D, E, F), 3,000 m (G, H, I), and 6,000 m (J, K, L) for the full distribution (A, D, G, J), critical habitat (B, E, H, K) and CHMS reserve (C, F, I, L) are shown.

**Figure 4:**
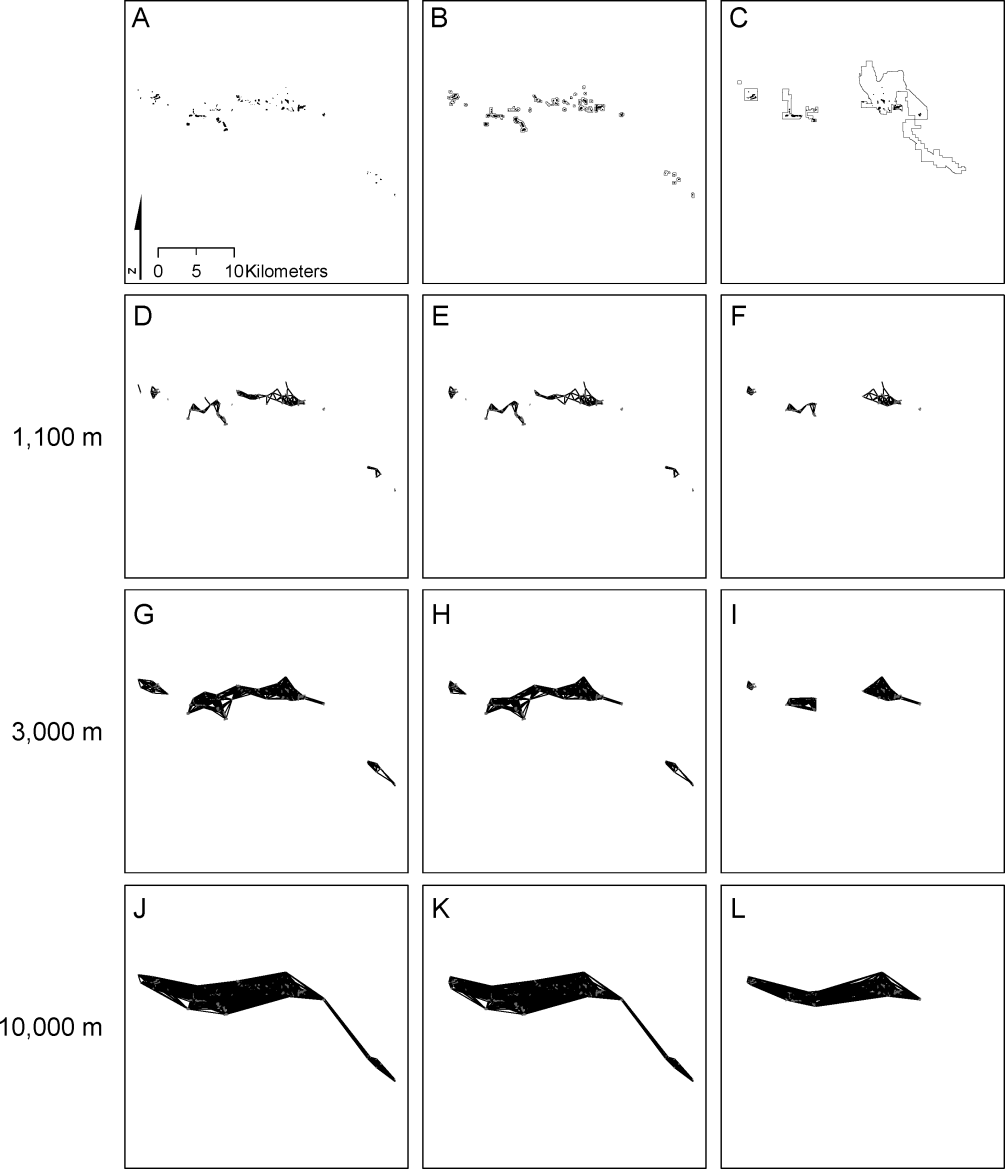
Distribution of and connectivity among *Acanthoscyphus parishii* var. *goodmaniana* patches. Full species distribution (A) compared with patches included in critical habitat (B) and the CHMS reserve (C). Edges connecting patches at distances of 1,100 m (D, E, F), 3,000 m (G, H, I), and 10,000 m (J, K, L) for the full distribution (A, D, G, J), critical habitat (B, E, H, K) and CHMS reserve (C, F, I, L) are shown.

The species we examine to demonstrate the approach are *Erigeron parishii* A. Gray (Asteraceae), *Eriogonum ovalifolium* var. *vineum* (Small) Jepson (Polygonaceae), and *Acanthoscyphus parishii* var. *goodmaniana* B. Ertter (Polygonaceae). These globally rare but locally common taxa (Rabinowitz, Cairns & Dillon, 1986) are primarily restricted to limestone and dolomite substrates in a ∼64,900 ha region (Fig. 1) on the arid northeastern slopes of the San Bernardino Mountains of southern California. Within their extremely small ranges the taxa naturally have patchily distributed high density occurrences totaling ∼220–∼550 ha (Table 2 and Figs. 2–4). Despite overlap in elevation and general habitat types (Gonella & Neel, 1995; Neel, 2000), they most often are not sympatric at specific sites. The primary threat that triggered listing under the ESA is anthropogenic habitat destruction and degradation from limestone mining (US Fish and Wildlife Service, 1994). They occur at more sites and have larger numbers of total individuals than many endangered plant species (Ellstrand & Elam, 1993; Neel, 2008a; Neel et al., 2012). Populations (typically with 100’s–1,000’s of individuals) are above sizes at which inbreeding and genetic drift are typically of extreme immediate concern (Ellstrand & Elam, 1993) and there is no evidence of intrinsic demographic decline within sites (US Fish and Wildlife Service, 1994). For such species, alleviating threats from habitat destruction and maintaining population distributions following the 3 R principles is likely to remove them from immediate danger of extinction or risk of being in such danger in the foreseeable future, as required for removing species from the ESA (16 USC. 1532 Sec. 3(3)).

**Table 2:** Size, extent, and connectivity of networks. Summary of number, size and connectivity measures for patches representing all known occurrences and occurrences included in critical habitat or the CHMS reserve for each of the three species: *Erigeron parishii*, *Eriogonum ovalifolium* var. *vineum*, and *Acanthoscyphus parishii* var. *goodmaniana*. Total area and patch sizes of patches that were excluded from the two conservation networks are given as well.

	*Erigeron*			*Eriogonum*			*Acanthoscyphus*		
Attribute	Full	Critical habitat	Forest service CHMS reserve	Full	Critical habitat	Forest service CHMS reserve	Full	Critical habitat	Forest service CHMS reserve
# of patches	86	72	50	227	202	112	97	89	52
Habitat area (ha)	444	409	297	548	517	326	218	210	129
Total excluded habitat area (ha)	N/A	29.04	147	N/A	27.88	222	N/A	8.38	75.52
Median area (ha) of patches in network	2.01	2.10^c^	1.67^d^	1.19	1.27^c^	**1.37** ^d^	0.94	1.03^c^	0.96^d^
Median area (ha) of patches excluded from network	N/A	**0.44** ^a^	1.91^b^	N/A	**0.52** ^a^	**0.80** ^b^	N/A	**0.61** ^a^	0.81^b^
Distance at which *NC* = 1 (km)	20.8	9.5	2.0	5.2	5.2	6.0	9.5	9.5	6.2
Maximum *EC(IIC*) (ha)	321.1	296.7	217.2	390.1	368.0	233.0	156.5	150.8	93.6
Distance at which maximum *EC(IIC)* is realized (km)	57.0	27.1	13.0	37.8	36.9	31.5	36.7	34.9	22.9
Minimum convex polygon area (ha)	26,506	12,325	5,368	28,005	25,445	15,548	19,484	18,917	7,520

**Notes.**

Boldface type indicates a significant difference based on Mann-Whitney U tests.

a Results of Mann–Whitney U tests for size of patches excluded from versus retained in the critical habitat patches: *Erigeron* *W* = 1598, *p* = 0.0002; *Eriogonum* *W* = 6155, *p* = 2.7 × 10^−8^; *Acanthoscyphus* *W* = 1209, *p* = 0.0007.

b Results of Mann–Whitney U tests for size of patches excluded from versus retained in the Forest Service CHMS: *Erigeron* *W* = 1384, *p* = 0.09; *Eriogonum* *W* = 9896, *p* = 9.5 × 10^−6^; *Acanthoscyphus* *W* = 1556, *p* = 0.25.

c Results of Mann–Whitney U tests for patch sizes of the full network versus the critical habitat network: *Erigeron* *W* = 2900.5748, *p* = 0.50; *Eriogonum* *W* = 21701, *p* = 0.43; *Acanthoscyphus* *W* = 4290.5, *p* = 0.94.

d Results of Mann–Whitney U tests for patch sizes of the full network versus the Forest Service CHMS network: *Erigeron* *W* = 2002, *p* = 0.51; *Eriogonum* *W* = 10994, *p* = 0.05; *Acanthoscyphus* *W* = 2409.5, *p* = 0.65.

As mentioned above, we examine two reserve networks for each of the three taxa. The first network is critical habitat established by the US Fish and Wildlife Service (2002b) (Figs. 2–4) that includes a subset of known occurrences of each species, but does not include buffering habitat. Because critical habitat provides essential life cycle needs (16 USC. 1532 Sec. 3(5)), it should reflect habitat minimally necessary for recovering species. The second network (the Carbonate Habitat Management Strategy, CHMS; Figs. 2–4) was developed by the Forest Service using a stakeholder-based planning process. It includes core reserves on National Forest land and Areas of Critical Environmental Concern (ACEC) on Bureau of Land Management land (S Eliason, San Bernardino National Forest, pers. commun., 2012). The CHMS reserve was designed to balance simultaneous conservation of all listed carbonate-endemic species and economic development by emphasizing sites with high quality plant habitat and low quality mineral ore. Including suitable but unoccupied habitat and unsuitable habitat between high density patches provides buffering from threats and maintenance of general ecological processes.

We present the first application of the full suite of graph metrics related to *IIC* to evaluating representation of network connectivity under the 3 Rs. This application allows objective evaluation of the complex phenomenon of fragmentation in the context of species status assessment and development of recovery criteria. Each metric provides insight into important, independent components of fragmentation that are confounded in most simple metrics. Addressing each landscape component in one integrated analysis provides managers with a practical and effective means of quantifying connectivity and network extent, thereby moving the 3 Rs beyond philosophical guidelines. By determining spatial scales at which structural connectivity changes, exactly how it changes, and by what magnitudes, managers can assess potential for biological risk or benefits of different scenarios for particular species. It can also be used to evaluate the effects of ecosystem-based conservation or surrogate species approaches on non-target species.

## Methods

### The taxa

*Erigeron parishii* is a low-growing, perennial herb that typically reaches 10–30 cm in height (Hickman, 1993). The 1–3 cm long, gray-green narrow leaves emerge in the spring and senesce by summer’s end, at which time the stems remain dormant above ground. Individual plants typically have many capitula with many lavender ray flowers and yellow disk flowers; each capitulum produces hundreds of 2–3 × 0.5 mm achenes. The 2–3 mm long pappus on each achene is unlikely to facilitate long-distance dispersal. Nothing is known about *Erigeron*’s mating system; everything from obligate, insect mediated outcrossing to agamospermy is known from the genus (Moldenke, 1976; Richards, 1999).

*Eriogonum ovalifolium* var. *vineum* is a long-lived, mound-forming herb to subshrub that grows as tall as 35 cm. Compact, spherical inflorescences 1.5–3 cm in diameter support 100’s of cream and magenta flowers and extend 3–6 cm above the vegetative portion of the plant (Hickman, 1993). Floral visitors include members of the Order Diptera in the families Bombyliidae, Chloropidae, Muscidae, Tachinidae, and Anthomyiidae as well as the family Halictidae in the Order Hymenoptera (M Neel & S Morita, 1998, unpublished data). Fruits are 2–3 mm long tear-drop shaped achenes that primarily arise from outcrossing (Neel, Ross-Ibarra & Ellstrand, 2001) and have no apparent specialized morphology to facilitate long-distance dispersal. Vegetative reproduction is possible but not confirmed (Neel, Ross-Ibarra & Ellstrand, 2001). Tepals become papery upon drying and tend to remain attached, potentially facilitating dispersal on the scale of 10s to possibly 100s of meters as dried inflorescence detach and blow along the surface of the ground by the wind.

*Acanthoscyphus parishii* var. *goodmaniana* is an annual with wiry, nearly leafless stems that rise 10–60 cm above a basal rosette of leaves. Inflorescences can comprise upwards of 50–100 min flowers with 3–4 mm long corollas that appear from June–September. There is no information on pollination biology, mating system, or dispersal. The ∼2 mm long achenes have no obvious dispersal adaptations.

### The landscapes

We started with all known patches that support high densities of each taxon as mapped by agency biologists through field survey (termed the full network). We then derived two networks of patches for each species that represent the conservation strategies. The first represents the subset of each full patch network that was designated as critical habitat (US Fish and Wildlife Service, 2002b). The second was the intersection between each full species network and the CHMS boundaries (provided by Scott Eliason, 2012, San Bernardino National Forest). We use the term patches to avoid confusion with the term occurrences, which itself is used by conservation organizations to avoid connotation of a biological population. In this context a patch is a discrete mapped polygon of occupied habitat, regardless of distances from other polygons. The combined area of patches is roughly equivalent to *Area of Occupancy* under IUCN Criterion B (Mace et al., 2008). There is no information on the degree to which patches function as populations or metapopulations although genetic data indicate relatively high gene flow (Neel & Ellstrand, 2001; Neel & Ellstrand, 2003).

Node and distance files were created with the program GenGraph using centroid to centroid Euclidean distance minus the patch radius of gyration (Urban, 2003). We used Euclidean distance because there exists no information with which to parameterize more sophisticated resistance surfaces. We weighted each patch by its area as calculated in ArcGIS Version 9.3 (Environmental Systems Research Institute, 2005). No information was available to weight patches by quality. Input files for GenGraph were created in ArcINFO 9.3 by using the GRID REGIONGROUP command to create rasters of patches of each species based on an eight neighbor rule and the GRID SAMPLE command to extract text representations of the rasters. Node and distance files from GenGraph were submitted to a command line version of Conefor v2.6 (Saura & Torné, 2012) with which we calculated all metrics for each network at 0.1 km increments across distance thresholds from 0.1 km to the point at which all patches form a single component in all three species (21 km) and also at the distance required to directly link all patches for each taxon. The former distance indicates the minimum dispersal distance that would be required for an organism to access all available habitat; the latter yields the maximum value of available habitat (*EC(IIC)*; see below) for a landscape.

### Quantifying networks

We described the size and extensiveness of networks of mapped habitat patches of the three species in terms of number and total area (*A*) and median area of patches, the distance at which all patches are linked into a single component, the distances at which all patches are directly linked to one another, and the size of the minimum convex polygon (MCP) that encompasses all patches. We quantified the number of components (*NC*) across a range of distances that yield complete isolation to complete connectivity. We used *IIC* to quantify connectivity as described below and in Table 1. *PC* could be used in the same manner but we prefer *IIC* because it accentuates threshold behavior and discontinuities that provide key insights into distances at which networks have structural vulnerability (Fig. S1). *IIC* was also found to better predict patterns of genetic diversity in another endemic plant species in the landscape we examined and thus may be a more appropriate indicator of connectivity (Neel, 2008b). *IIC* is a bounded connectivity index, ranging from zero to one, that increases with improved connectivity. *IIC* is scaled by total landscape area, such that when *IIC* = 1, the entire landscape is occupied by habitat (Pascual-Hortal & Saura, 2006). It can be expressed as an unbounded measure of the squared area of habitat (*IICnum*) or more effectively as quad equivalent connectivity (*EC(IIC)*), which is the square root of *IICnum* (Saura et al., 2011). *EC(IIC)* represents the size of a single habitat patch that would provide the same *IICnum* value as the observed habitat pattern. It reflects the amount of habitat effectively available to an organism based on size and configuration of patches and the organism’s ability to reach isolated patches. Values will be no smaller than the size of the single largest patch (Saura et al., 2011). When all patches are directly connected to one another by graph links (aka edges), *EC(IIC)* will fall short of the total habitat area as a function of the number of links required to connect patches. Although *EC(IIC)* is extremely useful, as can be seen in its calculation (Table 1), it confounds area and isolation. Fortunately these effects can be isolated by partitioning *IIC* into fractions and by comparing relative changes in total area versus *EC(IIC)* as described below.

Partitioning *IIC* starts with evaluating individual patch importance as the absolute change in *IICnum* of the network that results when each patch is left out (*varIIC*). We chose the absolute values of the *IIC* components (*var*) rather than the relative values (*delta*) because actual changes in area and connectivity are more informative and important in conservation than solely changes relative to amount of habitat in a network. *varIIC* is decomposed into three independent fractions of habitat availability: *varIIC_intra_*, *varIIC_flux_*, and *varIIC_connector_*. (Table 1; Baranyi et al., 2011; Bodin & Saura, 2010; Saura & Rubio, 2010). *varIIC_intra_* represents changes in connectivity conferred by area within individual patches, independent of any connections. *varIIC_flux_* quantifies connectivity of all pairs of patches in which the focal patch is a potential source or destination (i.e., is one end of a connected path) as a function of their areas and an inverse function of the number of links in the shortest path connecting them. *varIIC_connector_* quantifies changes in connectivity that result when loss of the focal patch eliminates a connection between two other patches or components. It is based on its location in the network and the sizes of the components it connects, not on the size of the focal patch itself. These fractions have been applied effectively to ranking the conservation value of individual patches based on their function in a landscape (Bodin & Saura, 2010). This one-at-a-time sensitivity analysis provides conservative estimates for network susceptibility because components that are connected through even just two patches would not be detected as vulnerable despite low redundancy. We used these fractions to quantify network sensitivity to landscape change by summing the values across all patches at each threshold distance yielding *sum_varIIC*and its fractions *sum_varIIC_intra_*, *sum_varIIC_flux_*, and *sum_varIIC_connector_*. We then quantified the percentage of total *sum_varIIC* contributed by each fraction (*thetaIIC_intra_*, *thetaIIC_flux_*, and *thetaIIC_connector_*).

The observed values were interpreted relative to the following general behavior of these metrics in landscapes. *sum_varIIC* increases with distance until all patches are directly connected to one another and thus all habitat in the landscape is reachable without stepping stones (Saura & Rubio, 2010). The overall increase in *sum_varIIC* is primarily due to the *sum_varIIC_flux_* fraction at all but the smallest distances. The absolute contribution of the *sum_varIIC_intra_* fraction is constant across distances, but has maximum relative contribution when patches are completely isolated and the only connectivity is within patches; this contribution drops rapidly as patches form components. Maximum *sum_varIIC_connector_* occurs at relatively short distances as components form through a single patch; secondary peaks at greater distances indicate initial coalescence of isolated components. These peaks occur where there is no redundancy in connections so loss of individual patches that break connections yields large differences in *IIC* values (Saura & Rubio, 2010).

We graphically assessed the magnitude of values of each fraction across distances, paying particular attention to distances at which the maximum absolute (*sum_varIIC*) and relative (*thetaIIC*) values of each fraction are achieved as well as the range of distances over which they show the greatest change in network values based on patch removal. The distances at which these changes occur indicate structural sensitivity that could affect potential for movement of species that disperse at or below these distances.

### Quantifying changes between networks

We used Mann–Whitney U tests to determine if the sizes of patches included in the reserve networks differed from patches that were excluded. We also used Mann–Whitney U tests to ask if the resulting patch size in reserves differed from size of patches in the full network. We quantified connectivity changes by comparing networks in terms of absolute and percentage differences for all measures described above (Table 1). Additionally, we compared rates of change in absolute area (*dA)* and effective connectivity (*dEC(IIC)*) (Table 1; Saura et al., 2011). Differences in these rates allow straightforward assessment of the degree to which changes are due to habitat loss alone versus due to additional effects of connectivity (Baranyi et al., 2011). If |*dEC*(*IIC*)| > |*dA*|, the change in habitat area substantially affects connectivity, typically through gain or loss of stepping stones that join otherwise isolated components as reflected by s*um_varIIC_connector_* (Saura et al., 2011). Conversely, when |*dEC*(*IIC*)| < |*dA*|, the habitat in question represents isolated patches that contribute to habitat connectivity only through the intrapatch area *sum_varIIC_intra_* (Saura et al., 2011). Finally, when |*dEC*(*IIC*)| ≈ |*dA*|, the subject habitat area is contiguous with the baseline habitat area and corresponds to small gains in connectivity reflected in the *sum_varIIC_intra_* and *sum_varIIC_flux_* (Saura et al., 2011).

Changes in all measures were also evaluated graphically to determine if distances at which patches would be structurally connected versus isolated or at which the three fractions of *varIIC* are important differed among networks. For example, if loss of stepping stone patches is indicated by a decrease in *varIIC_connector_*, commensurate relative changes in *varIIC_flux_* versus *varIIC_intra_* highlight how the loss of connectivity could affect landscape functioning by shifting potential for movement from among to within patches. This graphical analysis also allows detection of changes in the scales at which patches join into components. There is no single magnitude of a threshold or other change that is biologically important; rather it is incumbent upon the practitioner to interpret the pattern given the biological context of their system. Further, changes are not inherently good or bad; the consequences for the species or process must be evaluated individually. Even with this subjectivity, the results of these comparisons alert managers to the magnitude and scale of structural changes. The potential biological consequences then can be evaluated in the context of dispersal abilities of the species of interest or the spatial scales of the processes of interest. This information can be used to develop objective and measurable criteria related to representing connectivity (Table 1) that can be used in conjunction with range extent, habitat amount and ecological diversity to implement the 3 Rs. The graph metrics also provide a means of monitoring achievement of those criteria, or other changes in habitat status, as either conservation plan implementation or development result in altered landscape configuration.

## Results

### Existing networks

**Figure 5:**
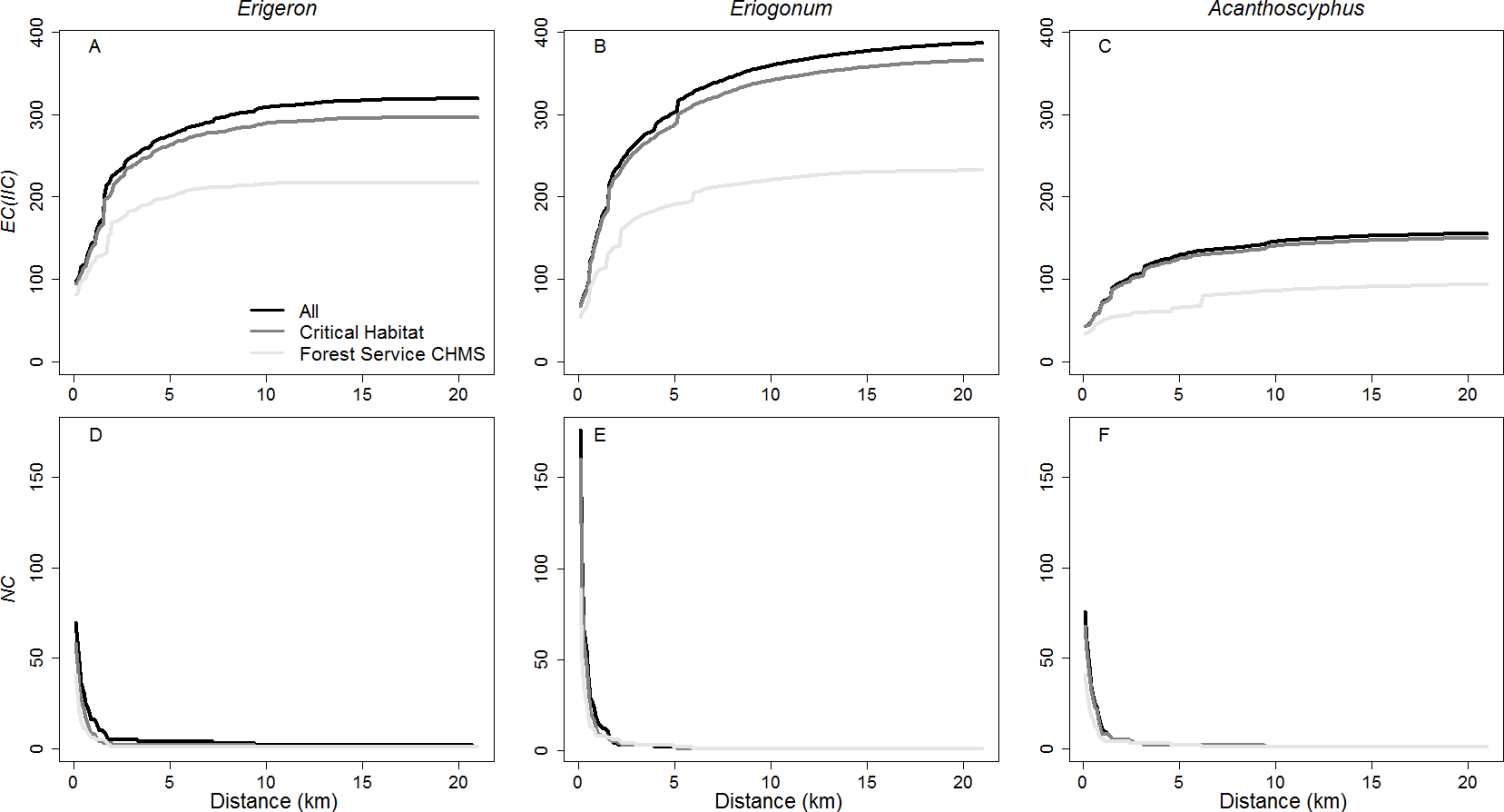
*EC(IIC)* and the number of components *(NC)* for networks. *EC(IIC)* (A–C) and the number of components (*NC*; D–F) across a range of distance thresholds for all *Erigeron parishii* (A, D), *Eriogonum ovalifolium* var. *vineum* (B, E), and *Acanthoscyphus parishii* var. *goodmaniana* (C, F) patches in the full network, critical habitat, and the Forest Service’s carbonate habitat management strategy.

*Erigeron* occupied 86 patches in a total of 444.2 ha (Fig. 2; Table 2). Mean and median patch area were 5.2 ha and 1.9 ha respectively (range = 0.04 ha-55.1 ha). At a distance of 20.8 km, all 86 patches comprised a single connected network with an *EC(IIC)* of 319.4 ha (Figs. 2 and 5). A maximum *EC(IIC)* of 321.1 ha was achieved at ∼57.0 km. When the network broke into 3 (at 9.4 km), 4 (at 8.3 km), and 5 (at 3.3 km) components, there were small but abrupt changes in *EC(IIC)* (Fig. 5). Generally though, *EC(IIC)* increased gradually above a distance of ∼3.5 km. There was a threshold at 1.7 km: above this distance >90% of patches were in one component; below it *NC* increased and *EC(IIC)* decreased dramatically (Fig. 5).

**Figure 6:**
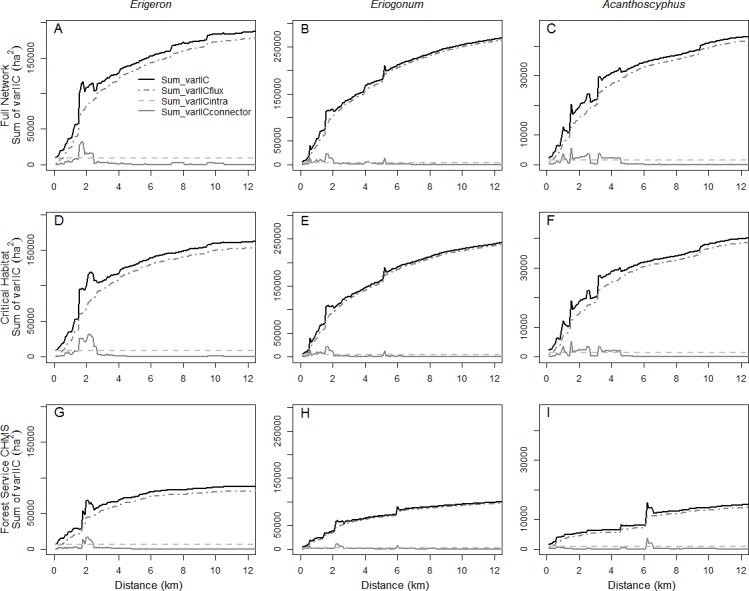
Absolute contributions of intrapatch area, flux, and connector fractions of connectivity. Values of total *sum_varIIC* and absolute values of *sum_varIICintra*, *sum_varIICflux*, and *sum_varIICconnector*, for the full network (A–C), critical habitat (D–F), and the Forest Service’s carbonate habitat management strategy (G–I) for *Erigeron parishii* (A, D, G), *Eriogonum ovalifolium* var. *vineum*, (B, E, H) and *Acanthoscyphus parishii* var. *goodmaniana* (C, F, I). Note that the *y*-axis values differ among species.

**Figure 7:**
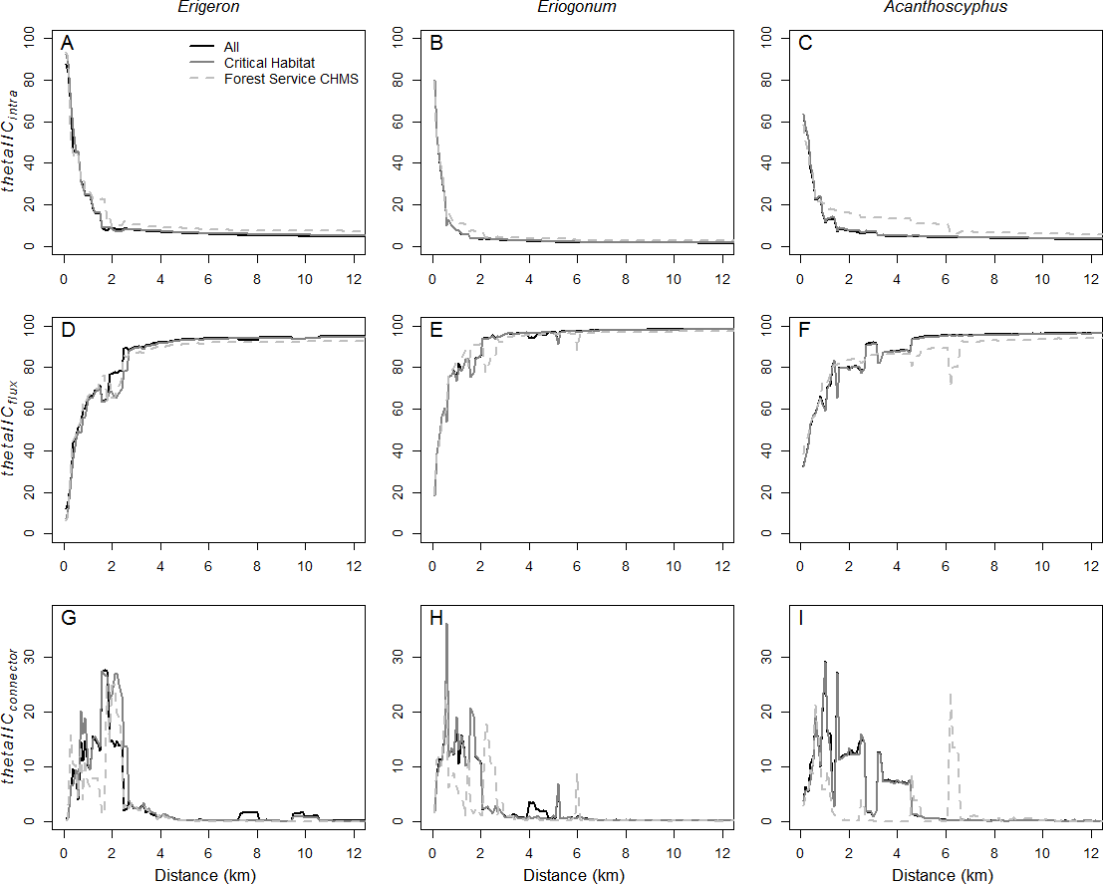
Relative contributions of intrapatch area, flux, and connector fractions of connectivity. Comparison of the relative (*theta*) contribution of *sum_varIICintra* (A–C), *sum_varIICflux* (D–F), and *sum_varIICconnector* (G–I) to *sum_varIIC* for the full network, critical habitat, and the Forest Service’s carbonate habitat management strategy for *Erigeron parishii* (A, D, G), *Eriogonum ovalifolium* var. *vineum*, (B, E, H) and *Acanthoscyphus parishii* var. *goodmaniana* (C, F, I). Note the y-axis scale difference for *thetaIICconnector*.

*sum_varIIC* followed expectations and increased with distance as all patches became increasingly directly connected to one another (Fig. 6). We observed one main discontinuity between 1.7 and 2.2 km and other minor ones at 7.3–8.3 km and 9.5–9.9 km (Fig. 6) coinciding with the *EC(IIC)* discontinuities noted above. These elevated values of *sum_varIIC* were due to *sum_varIIC_connector_*, including when it reached a maximum at 1.7–1.8 km (Fig. 6) at which point it comprised 28% of *sum_varIIC* (Fig. 7). Above the 2.5 km distance, *sum_varIIC_connector_* dropped to <1% of *sum_varIIC*, except at the 7.3–8.3 km and 9.5–9.9 km distances where it briefly increased to ∼1.5% of the total. These increases occurred at distances at which two multiple-component patches were joined through a single link. Above these distances *sum_varIIC_connector_* declined again as redundancy in connections increased (Figs. 2, 6 and 7). Component formation yielded commensurate increases in *sum_varIIC_flux_* (Fig. 6), which continues to increase with increasing redundancy in connections among patches, until it ultimately reached its maximum contribution of ∼95% (Fig. 7). Accordingly, *sum_varIIC_intra_* comprised 87% of the total *sum_varIIC* at the 0.1 km distance and declined rapidly to <10% by 1.6 km.

The 227 patches of *Eriogonum* occupied a total of 548 ha (Fig. 3; Table 2). Mean patch size was 2.4 ha and median area was 1.2 ha (range = 0.01 ha–25.2 ha). At a distance of ∼5.2 km, all patches comprised a single connected network with an *EC(IIC)* of 316.6 ha (Fig. 5). Maximum *EC(IIC)* (390.1 ha) occurred at a distance of ∼37.8 km. The transition from 1 to 2 components at 5.1 km yielded an abrupt change in *EC(IIC)* that represented 3.1% of the maximum value (Fig. 5); immediately above and below this distance, rates of change between 0.1 km distance steps were ∼0.1% indicating little change with distance. The network broke into 3 components at a distance of ∼3.9 km, but there was no large change in *EC(IIC)*. Below ∼2.1 km *EC(IIC)* declined and *NC* increased precipitously (Fig. 5).

*sum_varIIC* exhibited two main peaks corresponding to the component formation described above. Between 1.6 km and 2.1 km the contribution of *sum_varIIC_connector_* reached maximum (Fig. 6), comprising ∼20% of total *sum_varIIC* (Fig. 7). A smaller peak at which *sum_varIIC_connector_* increased to 6.6% of *sum_varIIC* resulted at 5.1 km (Fig. 7). At all other distances above the first threshold, *sum_varIIC_connector_* was <1% of *sum_varIIC* (Fig. 7). *sum_varIIC_intra_* comprised 80% of *sum_varIIC* at 0.1 km but rapidly declined to <10% by 0.9 km as the importance of *sum_varIIC_flux_* increased to its ultimate maximum contribution of ∼98.5% of the total (Fig. 7).

*Acanthoscyphus* patches occupied the smallest area, totaling only 217.9 ha in 97 patches (Fig. 4; Table 2). Mean patch area was 2.25 ha and median area was 0.98 ha (range = 0.03 ha–12.7 ha). *Acanthoscyphus* patches comprised a single component at a distance of ∼9.5 km at which point *EC(IIC)* was 130.3 ha (Figs. 4 and 5). Maximum *EC(IIC)* (156.5 ha) was achieved at a distance of 36.7 km. The network broke into 3 components at ∼3.1 km and into 7 components at ∼1.5 km (Fig. 5). As with *Erigeron*, *EC(IIC)*changed only slightly at these points but rapidly declined below ∼1.5 km (Fig. 5).

Change in *sum_varIIC* across distances in *Acanthoscyphus* was quite different than was seen in the other two species, with multiple peaks and declines between 1.0 and 4.2 km (Fig. 6). *sum_varIIC_connector_* also played a greater role over a larger range of distances (Figs. 6 and 7). It reached a maximum of ∼29% of the total *sum_varIIC* at 1 km (Fig. 7); a secondary peak at 1.5 km comprised 27% of the total and remained above 10% of *sum_varIIC* up to 2.6 km. Above that distance, *sum_varIIC_connector_* contributed a relatively constant 1–2% to total *sum_varIIC* (Fig. 7). *sum_varIIC_intra_* comprised 63% of the total *sum_varIIC* at the 0.1 km distance and, similar to *Erigeron*, contributed >10% of total *sum_varIIC* up to the 1.5 km distance. The relative contribution of *sum_varIIC_flux_* did not consistently exceed 90% until distances were ≥4.6 km; it finally reached its near-maximum contribution of ∼96% at 8.3 km (Fig. 7) at which point the contribution to *sum_varIIC_connector_* finally declined.

### Critical habitat networks

The critical habitat networks included ∼92–96.3% of the mapped area and maximum *EC(IIC)* for each taxon (Table 2). Patches of each species excluded from critical habitat were significantly smaller (median = 0.44 ha–0.61 ha) than included patches (median = 1.03 ha–2.10 ha; Table 2) and tended to be peripheral. Exclusion of small patches did not result in significant differences between patch size in the critical habitat versus full networks (Table 2). Range extents of *Eriogonum* and *Acanthoscyphus* as quantified by MCP were reduced by 8.1% and 2.9%, respectively, whereas MCP for *Erigeron* was reduced by 53.5% (Table 2).

The 72 critical habitat patches for *Erigeron* formed a single component at a threshold distance of 9.5 km. This >50% reduction relative to the full network (23.8 km; Table 2) resulted from exclusion of isolated patches in the southeastern portion of the range (Fig. 2). The 202 and 89 critical habitat patches for *Eriogonum* and *Acanthoscyphus* were connected into single components at distances that were identical to the full networks (5.2 km and 9.5 km, respectively).

**Figure 8:**
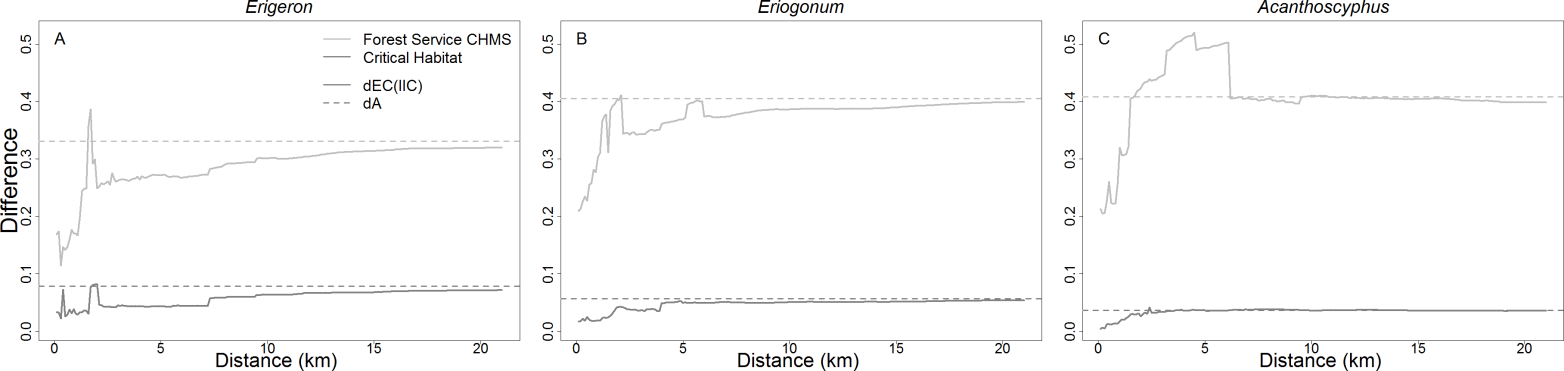
Relative change in area versus change in connectivity in reserve networks. Differences in rates of change in *dA* and *d(EC(IIC)* between the full network and the critical habitat and Forest Service carbonate habitat management strategy for *Erigeron parishii* (A), *Eriogonum ovalifolium* var. *vineum* (B), and *Acanthoscyphus parishii* var. *goodmaniana* (C).

As the critical habitat networks for the three species captured almost all existing habitat patches and the excluded patches were smaller and peripheral, *EC(IIC)* values were reduced by <10% of values in the existing networks (Fig. 8). Distances at which *EC(IIC)* declined precipitously and *NC* increased were the same as were observed in the full networks (Fig. 5). Overall, there were no fundamental differences in the three aspects of structural connectivity in that *sum_varIIC* fraction values for critical habitat deviated little from those in the full networks (Figs. 6 and 7). The only difference of note was that two thresholds found at 7.3–8.3 km and 9.5–9.9 km in *sum_varIIC_connector_* in the full *Erigeron* network were absent in critical habitat, indicating loss of connectivity via stepping stone patches at these distances (Figs. 2 and 6).

Reduction in *A* in critical habitat relative to full networks was greater than the commensurate reduction in *EC(IIC)* throughout almost all distances for each species (Fig. 8). *dEC(IIC)* was only slightly larger than *dA* at limited distance intervals. Thus, loss of habitat excluded from critical habitat would have relatively minor impacts to connectivity beyond reduction in area. Graphically comparing *thetaIIC_flux_* and *thetaIIC_intra_* between critical habitat and the full network confirms that connectivity comes primarily from these two aspects of connectivity (Fig. 7).

### Forest service (CHMS) networks

The CHMS networks included 59.3–66.8% of the area occupied by the full network for each species and 59.7–67.7% of the maximum *EC(IIC)* (Table 2). Median size of patches excluded from the CHMS ranged from 0.80 to 1.91 ha, whereas median size of included patches was 0.96–1.67 ha (Table 2). These differences in patch size were significant only for *Eriogonum*. Exclusion of smaller patches also resulted in significantly larger patch size in the CHMS relative to the full network for *Eriogonum* (Table 2). In addition to outright loss, some large *Erigeron* and *Acanthoscyphus* patches were only partially conserved or were broken into multiple patches (Figs. 2 and 4).

Exclusion of patches from both central and peripheral portions of each species’ range (Figs. 2–4) affected network extent and patch isolation. The distance required to connect the 50 CHMS *Erigeron* patches into a single component (2.0 km) was ∼10% of what was required in the full network and the MCP was 20% of the full network value (Table 2). Network extensiveness of the 52 *Acanthoscyphus* patches was also reduced in that the distance required to form one component was 65% of the original (6.2 km) and MCP was 38.5% of the original size (Table 2). By contrast, due to loss of centrally located patches the distance required for the 112 *Eriogonum* patches in the CHMS to form a single component was greater than in the full network (6.0 vs. 5.2 km; Table 2 and Fig. 5) even though the overall extent as measured by maximum distance between patches declined from 36.8 km to 31.5 km (Table 2) and MCP was reduced by 44.5%.

*EC(IIC)* values for the CHMS networks were 20–52% of those in the full species networks at all distances (Fig. 8). As with the full *Erigeron* and *Eriogonum* networks, below a distance of ∼1.5–2.5 km *EC(IIC)* declined and *NC* increased precipitously (Fig. 5). For *Acanthoscyphus* the decrease in *EC(IIC)* at smaller distances was more gradual and a discontinuity that was not observed in the full network was noted at 6.2 km where all patches formed one component.

The curves for overall *sum_varIIC* for the *Erigeron* and *Eriogonum* CHMS networks were necessarily of lower magnitude than the full networks due to smaller starting area, but the general patterns of increase with distance were the same (Fig. 6). By contrast, the *sum_varIIC* curve for *Acanthoscyphus* differed in both shape and magnitude, indicating fundamental changes in structural connectivity (Figs. 6 and 7).The *sum_varIIC_connector_* fraction (Fig. 6) was greatly affected for all species, but as described below specific effects differed. For *Erigeron,* the magnitude was lower in the CHMS landscape but maximum values occurred at approximately the same threshold distance (∼2 km; Fig. 6). In *Eriogonum*, the peaks in *sum_varIIC_connector_* at 1–2 km in the full landscape were replaced by a much smaller peak at >2 km in the CHMS network (Fig. 6). Differences in *sum_varIIC_connector_* for *Acanthoscyphus* were greater than for the other two species; the connector fraction in the full landscape was important at distances occurring from 0.5–4 km, whereas in the CHMS network it was not important until ∼6.2–6.5 km. Changes at these distances yielded greater increases in *thetaIIC_intra_* than in *thetaIIC_flux_* (Fig. 7) indicating that loss of stepping-stone patches at distances <6 km results in isolation of individual patches and smaller components over greater distances than in the full network.

*dEC(IIC)* values across distance thresholds indicated that effective habitat availability in the CHMS networks deviated dramatically from the full networks (Fig. 8). For *Erigeron* the deviation exhibited a sharp peak of 38% at 1.7 km and was ∼25–30% throughout most of the distance range. *Eriogonum* reached a maximum 41% deviation at 2.1 km, and values remained above 38% at distances >5 km. *dEC(IIC)* for *Acanthoscyphus* reached a maximum deviation of 52% at distances of 3.2–6.2 km.

The reduction in *EC(IIC)* in the CHMS relative to full networks (*dEC(IIC)*) was less than the corresponding decrease in area (*dA*) throughout most of the range of distances for *Erigeron* and *Eriogonum*. Exceptions occurred at distances at which *dEC(IIC)* reached its maximum (1.6–1.7 km for *Erigeron* and at 2.1 km for *Eriogonum*; Fig. 8). For *Acanthoscyphus dEC(IIC)* exceeded *dA* at distances of 1.7–6.1 km (Fig. 8). Thus, changes are primarily due to habitat loss for *Eriogonum* and *Erigeron*, but are due more to changes in connectivity among patches in *Acanthoscyphus*. This differential effect of area versus connectivity can also be seen in the differences in *thetaIIC_flux_* and *thetaIIC_intra_* between the CHMS and full networks (Fig. 7).

## Discussion

Ultimately, species conservation requires maintaining enough individuals in stable or increasing populations such that extinction risks are low enough to ensure persistence. Conservation assessment and planning based on individual abundances is hindered by lack of detailed data on population sizes and dynamics (Mace et al., 2008) and error in risk assessment is higher when data are limited (Wilson, Kendall & Possingham, 2011). The 3 Rs (Shaffer & Stein, 2000) offer an approach to conservation that embodies basic conservation science principles (Soulé & Simberloff, 1986), that species persistence fundamentally depends on maintaining habitat in amounts and configurations that sustain ecological and evolutionary processes. These principles have solid scientific foundation (Noss, O’Connell & Murphy, 1997) and are the basis for IUCN status assessments (He, 2012; Mace et al., 2008). As such, data on habitat amount and distribution have great potential for use in species assessments (Attorre et al., 2013) and in conservation planning, more generally. But quantitative assessment of the 3 Rs has been limited to counting numbers of sites or area available for different elements of diversity, and changes in connectivity have been particularly difficult to incorporate effectively. We have demonstrated a flexible graph theoretic method to quantify habitat availability conferred by the landscape structure and detect the magnitude of changes when that structure is altered. When integrated with representation of the ecological and geographic range of a species, these resulting measures yield a practical means of applying the 3-R guidelines. This method enables assessing conservation status and establishing objective and measureable recovery criteria even when data are limited, and provides a complementary approach when individual abundances and population trajectories are also available. Moreover, it provides a means of evaluating the species-specific effects of ecosystem-based or surrogate species approaches.

### Quantifying networks

In examining the full suite of graph metrics derived from *IIC* in three species, we have demonstrated the generality of trends in behavior of the metrics previously noted (Bodin & Saura, 2010; Saura & Rubio, 2010). At the same time, we highlight their ability to distinguish species-specific connectivity patterns that indicate potential differences in ecological functioning and sensitivities to changes in structure that can inform setting species specific recovery criteria. Further, we showed the importance of examining multiple metrics to gain insight into different aspects of structure that can be affected. For all species, patterns of increase in *EC(IIC)* with distance indicate the degree to which patches in a landscape network are accessible as a function of dispersal abilities (Fig. 5). Differences in magnitude of *EC(IIC)* among species and networks appropriately reflect differences in overall habitat amount. Relatively steep slopes in *EC(IIC)* at distances <1–2 km indicate rapid increase in habitat accessibility with increasing dispersal ability as patches coalesce into local multi-patch components. Above distances at which this coalescence first occurs, *EC(IIC)* continues to increase as there is redundancy in connections within components and patches become linked directly rather than indirectly through other patches. Abrupt changes in *EC(IIC)* reflect key transitions in habitat availability as components that were isolated at smaller threshold distances become linked; the fractions of *sum*_*varIIC* identify exactly how different aspects of connectivity are affected at these transitions. Of particular interest are transient increases in the connector fraction (*sum_varIIC_connector_*) because they indicate connections among components through single patches. Above and below the transition points, the relative importance of flux (*sum_varIIC_flux_*) versus intrapatch (*sum_varIIC_intra_*) fractions provides insight into the degree to which movement is facilitated with extensive contiguous habitat versus among habitat patches that have multiple connections. Smooth increases in *EC(IIC)* (Fig. 5) throughout most of the gradient for *Eriogonum* and *Erigeron* with few small discontinuities indicated that patches were more or less evenly spaced and coalesced into components incrementally with increasing distances; the patterns in *sum_varIIC* supported this interpretation (Figs. 6 and 7). Although the *EC(IIC)* curve for *Acanthoscyphus* looked similar to curves for the other species, *sum_varIIC* showed many more abrupt discontinuities. These repeated abrupt changes at specific distances indicate that patches of *Acanthoscyphus* were more locally clustered into components that had internally redundant connections, but that remained isolated from other components for greater distances. The distances at which those components connect to one another are indicated by large jumps in *sum_varIIC*. The greater importance of *sum_varIIC_connector_* over a much larger range of threshold distances for *Acanthoscyphus* indicates more connections among components via single links than we saw for *Erigeron* and *Eriogonum* (Fig. 7). In summary, the natural distribution of *Acanthoscyphus* has more isolated clusters of patches with less total area whereas *Erigeron* and *Eriogonum* patches are more regularly spaced. These differences among species then interact with the spatial distribution of the reserves as detailed below.

### Quantifying changes between networks

Critical habitat networks mostly met expectations for the 3-R principles in that they represented >90% of high density patches and resulted in minimal deviations from patterns of potential connectivity in the existing networks as measured by *EC(IIC)* and *sum_varIIC* (Table 2, Figs. 5–8). Excluded patches were primarily small and isolated and were often from the periphery of each taxon’s range. The most substantial impact was the ∼50% reduction in the spatial extent of the *Erigeron* network resulting from exclusion of the few, small, patches isolated in the southeast portion of the range. Although this range reduction is of concern for representing the species ecological and geographic range, effects on connectivity and habitat availability *per se* were minimal. For all species, habitat availability remained within 10% of the full network values (Table 1), differences were primarily due to habitat loss (Fig. 8), and distances at which most major abrupt changes were seen were mostly the same as in the full network. The most notable difference in thresholds was for *sum_varIIC_connector_* for *Erigeron* at distances between 7 and 10 km, where loss of patches eliminated components that were previously joined by stepping-stone links in the full network (Figs. 2 and 6). It is unlikely that this loss would strongly affect species connectivity because dispersal at this spatial scale is highly improbable given lack of long distance dispersal mechanisms for the achenes of this species.

By contrast, persistence of all three species would potentially be compromised under the CHMS due to exclusion of >50% of the patches and 36–41% of total habitat area. The excluded patches were both peripherally and centrally located and their loss would change both the spatial extents of networks and the scales at which they break into different components (Figs. 2–5). As with critical habitat, reduction in network extensiveness due to loss of peripheral patches was greatest for *Erigeron* (with the distance required for all patches to form fully connected networks being 10% and MCP being 20% of the full network). Although it may at first be counterintuitive, the combination of loss of central and peripheral patches under the CHMS increased the distance required to connect *Eriogonum* patches into a single component from 5.2 km to 6.0 km (Fig. 5), whereas distances required to directly connect all patches decreased from 37.8 km to 31.5 km and MCP decreased by >12,500 ha (Table 2). Changes in the shapes of the *sum_varIIC* curves, distances at which key thresholds are located, and relative contributions of the *sum_varIIC* fractions (Figs. 6 and 7), document the risk of potentially altering ecological processes due to changing landscape structure. Although all species were affected, *dA* and *dEC(IIC)* values indicate that habitat loss had a greater additional effect on connectivity for *Acanthoscyphus* than for *Erigeron* or *Eriogonum* (Fig. 8).

Differential effects across these three species illustrate utility of the metrics in evaluating planning approaches that seek to conserve many species simultaneously or to develop plans using surrogate species approaches. Even though they are ecologically similar and are restricted to similar geologic substrates in the same landscape, effects of the same reserve network varied tremendously due to the intersection between the reserve boundaries and the distribution of each species. Using this graph approach to assess the species-specific effects of the proposed multi-species conservation plan based on commonly available distribution maps can show where modifications are needed to ensure sufficiency for all species rather than naively assuming plans based on one surrogate species are adequate for all others. Thresholds or discontinuities based on landscape structure indicate potential change in functioning of ecological processes; actual risk depends on whether changes coincide with dispersal capabilities of targeted species. Optimal application of graph theory to species assessment and criteria development requires understanding how changes in structural connectivity affect functional connectivity. Links between structural and functional connectivity depend on complex interactions between landscape pattern and dispersal abilities of particular species (Saura et al., 2011). Thus knowledge of dispersal is key to integrating landscape pattern with ecological and evolutionary processes to inform decision making. Increasing use of DNA-based approaches to measuring movement (e.g., Broquet & Petit, 2009) is improving knowledge of actual dispersal.

However, for most species, including the three we investigated here, dispersal capabilities remain unknown. To accommodate such uncertainty related to this knowledge gap, we quantified potential connectivity across a range of distances that exceed the reasonable range of dispersal potential. We then compared the distances that show potential susceptibility to dispersal distances for similar species to inform a basic risk assessment (e.g., Lookingbill et al., 2010). Such general analyses can be used to justify minimizing changes at key high-risk distances or to recommend conducting research to better determine actual scales of dispersal if uncertainty is considered too great to commit land or funding without additional information. For example, in general, seed dispersal >1 km is unlikely for plant species with no specialized dispersal mechanism (Cain, Damman & Muir, 1998; Clark et al., 2005; Greene & Johnson, 1996; Hardy et al., 2006), although longer distance dispersal is possible (Cain, Milligan & Strand, 2000). Similarly, pollen flow distances are typically <1 km for wind-dispersed pollen (Ellstrand, 2003) and 500–1,700 m for insect-pollinated plants (Williams et al., 2007). As a result, structural landscape changes that occur at distances of <∼1–2 km are of greatest concern due to the risk that changes in connections could alter the landscape such that dispersal goes from possible to impossible. Even the full existing networks for the species we examined were not fully connected at these distances, although they were locally connected into smaller components that yielded effective connectivity of *EC(IIC)* = 96–235 ha at 2 km (Figs. 2–4). Unfortunately, it is exactly in the 1–2 km range that all species networks were most vulnerable to loss of stepping stone patches (as indicated by maximum values of *sum_varIIC_connector_*; Fig. 7). Further, the CHMS excluded patches that conferred stepping stone functions at these distances for all species although the magnitude of potential effects varied by species. The *EC(IIC)* values at 2 km represented in CHMS reserves ranged from 55–169 ha, indicating retention of 56–75% of the *EC(IIC)* of the full networks at this distance. Vulnerability in connectivity for the CHMS reserves based on *dEC(IIC)* exceeding *dA* was minor for *Erigeron*and *Eriogonum* and occurred at distances around the 1 km threshold (Fig. 8). Vulnerability was much greater for *Acanthoscyphus* over a larger range of distances (from 1.7 to 6.1 km; Fig. 8). Because components that are naturally >∼2–3 km apart are not likely exchanging migrants on a regular basis, even without anthropogenic habitat loss, we suggest additional conservation effort for patches that maintain connections within components that form at ∼1–2 km is a high priority for this species.

### Conservation criteria

The graph metrics offer an effective means of comprehensively assessing habitat availability based on amount and spatial configuration in landscapes and quantifying change resulting from losing or gaining habitat area. They allow moving beyond vague descriptions of fragmentation and unmeasurable conservation goals such as ‘maintaining natural levels of connectivity’ to establishing quantitative criteria (Table 1) that help bring the 3 Rs into a quantitative framework. For example, managers could establish recovery criteria that maintain habitat availability within a given percentage of a chosen baseline (e.g., current or historical conditions) through conservation or restoration. Criteria could also be stated in terms of maintaining specific aspects of connectivity such as the flux or connector fractions of *sum_varIIC* as is relevant to a species of interest. Graph analyses also provide a means of assessing if criteria are met by quantifying absolute or relative change in effective habitat availability that would result from alternative conservation plans, projected future conditions, or management actions. Beyond simply highlighting amounts of habitat, it is easy to identify distance thresholds at which different aspects of structural connectivity are most sensitive to change. Affirming that habitat availability has been altered by less than a given percentage, or limiting change in *sum_varIIC_connector_* at a key dispersal distances that would result from loss of stepping stone patches provides a much stronger basis for evaluating risk than simply saying habitat is fragmented. Management actions that target protecting key connections or adding redundancy to a network through restoration can be specified. Further, the specific locations in a landscape that would accomplish this goal can be identified.

Despite the advances offered by this comprehensive approach to quantifying habitat availability, there is still no prescribed “amount” of connectivity that is necessary or sufficient for all species (which has also been pointed out by others; e.g., Saura et al., 2011). And the interpretation of which thresholds and changes are large enough to be of concern is subjective. Although there is strong scientific foundation for the importance of maintaining habitat area and quality, as with other types of thresholds, the exact amount needed or degree of change deemed acceptable is ultimately based on normative values (Robbins, 2009; Tear et al., 2005; Vucetich, Nelson & Phillips, 2006). As such, practitioners must evaluate what degree of change is acceptable for a given context. As a result, quantitative criteria based on these analyses could suffer from the same criticisms of subjectivity as existing population and individual recovery criteria (Elphick, Reed & Bonta, 2001; Neel & Che-Castaldo, 2013; Neel et al., 2012). Still, we argue that they provide improved means for making transparent decisions, the basis of which can then be discussed and debated. One way to reduce the subjective nature of exact thresholds would be to translate habitat availability into an estimated probability of persistence or extinction (Robbins, 2009). In this way different habitat availability thresholds for different species could be compared in terms of a common currency. Potential methods applicable to quantifying extinction risk at multiple sites given the sizes and isolation of the constituent patches include Hanski’s (1994) incidence function (the probability each patch will be occupied at equilibrium) and metapopulation persistence capacity (the ability of a landscape to support persistence of a metapopulation; Hanski & Ovaskainen, 2000; Ovaskainen & Hanski, 2001). Effective area could also potentially be linked to rate of change in population size through posited relationships between habitat area and individual abundance (He, 2012); the generality of these relations warrant further investigation and development.

In summary, the approach we demonstrate here is broadly useful for evaluating the consequences of environmental changes or management actions that alter landscape pattern (e.g., habitat loss, conservation, or restoration). Because alterations to habitat amount and connectivity will likely confer changes in ecological processes in landscapes, maintaining amounts of habitat or populations is a fundamental conservation goal and basis for assessing change in conservation status. The approach provides an effective means of making scientifically defensible decisions based on potential risk to species even if few data are available, and allows additional data to be incorporated if they become available. It is well suited to evaluating alternative strategies and to monitoring decline or progress towards attainment of recovery criteria. More generally, it provides the means to quantify many aspects of resiliency that have previously been lacking.

## Supplemental Information

10.7717/peerj.622/supp-1 Figure S1 *EC(PC)* as a function of distance EC(PC) across a range of distance thresholds for all *Erigeron parishii* (A), *Eriogonum ovalifolium* var. *vineum* (B), and *Acanthoscyphus parishii* var. *goodmaniana* (C) patches in the full network, critical habitat, and the Forest Service’s carbonate habitat management strategy. *PC* was calculated with a 0.25 probability of dispersing at each selected threshold distance. *EC(IIC)* in the full landscape for each taxon is given for comparison. At the shortest distances *EC(PC)* was within 0.2–2% of *EC(IIC)* for full landscapes. *EC(IIC)* values then increase more rapidly at intermediate distances but maximum values of *EC(PC)* were ∼11% larger than *EC(IIC)* for *Eriogonum* and *Acanthoscyphos*, but differed by less than 1% for *Erigeron*. These relative values will vary with different probabilities of dispersal in *PC*.

10.7717/peerj.622/supp-2 Supplemental Information 2 Raw graph metric values for all landscapes

## References

[ref-1] Attorre F, De Sanctis M, Farcomeni A, Guillet A, Scepi E, Vitale M, Pella F, Fasola M (2013). The use of spatial ecological modelling as a tool for improving the assessment of geographic range size of threatened species. Journal for Nature Conservation.

[ref-2] Baranyi G, Saura S, Podani J, Jordan F (2011). Contribution of habitat patches to network connectivity: redundancy and uniqueness of topological indices. Ecological Indicators.

[ref-3] Bengtsson J, Angelstam P, Elmqvist T, Emanuelsson U, Folke C, Ihse M, Moberg F, Nystrom M (2003). Reserves, resilience and dynamic landscapes. Ambio.

[ref-4] Bodin O, Saura S (2010). Ranking individual habitat patches as connectivity providers: integrating network analysis and patch removal experiments. Ecological Modelling.

[ref-5] Broquet T, Petit EJ (2009). Molecular estimation of dispersal for ecology and population genetics. Annual Review of Ecology Evolution and Systematics.

[ref-6] Butchart SHM, Walpole M, Collen B, van Strien A, Scharlemann JPW, Almond REA, Baillie JEM, Bomhard B, Brown C, Bruno J, Carpenter KE, Carr GM, Chanson J, Chenery AM, Csirke J, Davidson NC, Dentener F, Foster M, Galli A, Galloway JN, Genovesi P, Gregory RD, Hockings M, Kapos V, Lamarque JF, Leverington F, Loh J, McGeoch MA, McRae L, Minasyan A, Morcillo MH, Oldfield TEE, Pauly D, Quader S, Revenga C, Sauer JR, Skolnik B, Spear D, Stanwell-Smith D, Stuart SN, Symes A, Tierney M, Tyrrell TD, Vie JC, Watson R (2010). Global biodiversity: Indicators of recent declines. Science.

[ref-7] Cain ML, Damman H, Muir A (1998). Seed dispersal and the Holocene migration of woodland herbs. Ecological Monographs.

[ref-8] Cain ML, Milligan BG, Strand AE (2000). Long-distance seed dispersal in plant populations. American Journal of Botany.

[ref-9] Calabrese JM, Fagan WF (2004). A comparison shoppers’ guide to connectivity metrics: trading off between data requirements and information content. Frontiers in Ecology and the Environment.

[ref-10] Clark CJ, Poulsen JR, Bolker BM, Connor EF, Parker VT (2005). Comparative seed shadows of bird-, monkey-, and wind-dispersed trees. Ecology.

[ref-11] Ellstrand NC (2003). Current knowledge of gene flow in plants: implications for transgene flow. Philosophical Transactions of the Royal Society of London Series B.

[ref-12] Ellstrand NC, Elam DR (1993). Population genetic consequences of small population size: implications for plant conservation. Annual Review of Ecology and Systematics.

[ref-13] Elphick CS, Reed JM, Bonta JM (2001). Correlates of population recovery goals in endangered birds. Conservation Biology.

[ref-14] Environmental Systems Research Institute (2005).

[ref-15] Fahrig L (1997). Relative effects of habitat loss and fragmentation on population extinction. Journal of Wildlife Management.

[ref-16] Fahrig L (2002). Effect of habitat fragmentation on the extinction threshold: a synthesis. Ecological Applications.

[ref-17] Ferrari JR, Lookingbill TR, Neel MC (2007). Two measures of landscape-graph connectivity: assessment across gradients in area and configuration. Landscape Ecology.

[ref-18] Frankham R (2005). Conservation biology—ecosystem recovery enhanced by genotypic diversity. Heredity.

[ref-19] Gonella MP, Neel MC, Roundy BA, McArthur ED, Haley JS, Mann DK (1995). Characterization of rare plant habitat for restoration in the San Bernardino national forest.

[ref-20] Greene DF, Johnson EA (1996). Wind dispersal of seeds from a forest into a clearing. Ecology.

[ref-21] Hanski I (1994). A practical model of metapopulation dynamics. Journal of Animal Ecology.

[ref-22] Hanski I, Ovaskainen O (2000). The metapopulation capacity of a fragmented landscape. Nature.

[ref-23] Hanski I, Simberloff D, Hanski I, Gilpin ME (1996). The metapopulation approach, its history, conceptual domain, and application to conservation. Metapopulation biology.

[ref-24] Hardy OJ, Maggia L, Bandou E, Breyne P, Caron H, Chevallier MH, Doligez A, Dutech C, Kremer A, Latouche-Halle C, Troispoux V, Veron V, Degen B (2006). Fine-scale genetic structure and gene dispersal inferences in 10 neotropical tree species. Molecular Ecology.

[ref-25] He FL (2012). Area-based assessment of extinction risk. Ecology.

[ref-26] Hickman JC (1993). The Jepson manual: higher plants of California.

[ref-27] Holling CS (1973). Resilience and stability of ecological systems. Annual Review of Ecology and Systematics.

[ref-28] IUCN (2010). Guidelines for using the IUCN red list categories and criteria.

[ref-29] Jagers P, Harding KC (2009). Viability of small populations experiencing recurring catastrophes. Mathematical Population Studies.

[ref-30] Leidner AK, Neel MC (2011). Taxonomic and geographic patterns of decline for threatened and endangered species in the United States. Conservation Biology.

[ref-31] Lookingbill TR, Gardner RH, Ferrari JR, Keller CE (2010). Combining a dispersal model with network theory to assess habitat connectivity. Ecological Applications.

[ref-32] Looy K, Cavillon C, Tormos T, Piffady J, Landry P, Souchon Y (2013). A scale-sensitive connectivity analysis to identify ecological networks and conservation value in river networks. Landscape Ecology.

[ref-33] Mace GM, Collar NJ, Gaston KJ, Hilton-Taylor C, Akcakaya HR, Leader-Williams N, Milner-Gulland EJ, Stuart SN (2008). Quantification of extinction risk: IUCN’s system for classifying threatened species. Conservation Biology.

[ref-34] Margules CR, Pressey RL (2000). Systematic conservation planning. Nature.

[ref-35] McGarigal K, Cushman SA, Neel MC, Ene E (2002). FRAGSTATS: spatial pattern analysis program for categorical maps.

[ref-36] Moldenke AR (1976). California pollination ecology and vegetation types. Phytologia.

[ref-37] National Marine Fisheries Service (2010). Interim endangered and threatened species recovery planning guidance.

[ref-38] Neel MC (2000). The structure of diversity: implications for reserve design. Ph.D. diss..

[ref-39] Neel MC, Carroll SP, Fox CW (2008a). Conservation planning and genetic diversity. Conservation biology: evolution in action.

[ref-40] Neel MC (2008b). Patch connectivity and genetic diversity conservation in the federally endangered and narrowly endemic plant species A*stragalus albens* (Fabaceae). Biological Conservation.

[ref-41] Neel MC, Che-Castaldo JP (2013). Do past abundances or biological traits predict recovery objectives for threatened and endangered plant species?. Conservation Biology.

[ref-42] Neel MC, Ellstrand NC (2001). Patterns of allozyme diversity in the threatened plant *Erigeron parishii* (Asteraceae). American Journal of Botany.

[ref-43] Neel MC, Ellstrand NC (2003). Conservation of genetic diversity in the endangered plant *Eriogonum ovalifolium* var. *vineum* (Polygonaceae). Conservation Genetics.

[ref-44] Neel MC, Leidner A, Haines A, Goble DD, Scott JM (2012). By the numbers: how is recovery defined by the US Endangered Species Act?. Bioscience.

[ref-45] Neel MC, McGarigal K, Cushman SA (2004). Behavior of class-level landscape metrics across gradients of class aggregation and area. Landscape Ecology.

[ref-46] Neel MC, Ross-Ibarra J, Ellstrand NC (2001). Implications of mating patterns for conservation of the endangered plant *Eriogonum ovalifolium* var. *vineum* (Polygonaceae). American Journal of Botany.

[ref-47] Noss RF, O’Connell MA, Murphy D (1997). The science of conservation planning: habitat conservation under the endangered species act.

[ref-48] Ovaskainen O, Hanski I (2001). Spatially structured metapopulation models: global and local assessment of metapopulation capacity. Theoretical Population Biology.

[ref-49] Pascual-Hortal L, Saura S (2006). Comparison and development of new graph-based landscape connectivity indices: towards the prioritization of habitat patches and corridors for conservation. Landscape Ecology.

[ref-50] Rabinowitz D, Cairns S, Dillon T, Soulé ME (1986). Seven forms of rarity and their frequency in the flora of the British Isles. Conservation biology: the science of scarcity and diversity.

[ref-51] Richards A (1999). Plant breeding systems.

[ref-52] Robbins K (2009). Strength in numbers: setting quantitative criteria for listing species under the Endangered Species Act. UCLA Journal of Environmental Law and Policy.

[ref-53] Rubio L, Saura S (2012). Assessing the importance of individual habitat patches as irreplaceable connecting elements: an analysis of simulated and real landscape data. Ecological Complexity.

[ref-54] Saura S, Estreguil C, Mouton C, Rodriguez-Freire M (2011). Network analysis to assess landscape connectivity trends: application to European forests (1990–2000). Ecological Indicators.

[ref-55] Saura S, Pascual-Hortal L (2007). A new habitat availability index to integrate connectivity in landscape conservation planning: comparisons with existing indices and application to a case study. Landscape and Urban Planning.

[ref-56] Saura S, Rubio L (2010). A common currency for the different ways in which patches and links can contribute to habitat availability and connectivity in the landscape. Ecography.

[ref-57] Saura S, Torné J (2012).

[ref-58] Shaffer ML, Stein BA, Stein BA, Kutner LS, Adams JS (2000). Safeguarding our precious heritage. Precious heritage: the status of biodiversity in the United States.

[ref-59] Simberloff D (1998). Flagships, umbrellas, and keystones: is single-species management passe in the landscape era?. Biological Conservation.

[ref-60] Soulé ME, Simberloff D (1986). What do genetics and ecology tell us about the design of nature reserves?. Biological Conservation.

[ref-61] Stacey PB, Taper M (1992). Environmental stochasticity and the persistence of small populations. Ecological Applications.

[ref-62] Tear TH, Kareiva P, Angermeier PL, Comer P, Czech B, Kautz R, Landon L, Mehlman D, Murphy K, Ruckelshaus M, Scott JM, Wilhere G (2005). How much is enough? The recurrent problem of setting measurable objectives in conservation. Bioscience.

[ref-63] US Fish and Wildlife Service (1994). Endangered and threatened wildlife and plants: final rule. Five plants from the San Bernardino Mountains in southern California determined to be threatened or endangered. 50 CFR Part 17. Federal Register.

[ref-64] US Fish & Wildlife Service (2002a). Recovery plan for gabbro soil plants of the central Sierra Nevada foothills.

[ref-65] US Fish and Wildlife Service (2002b). Designation of critical habitat for five carbonate plants from the San Bernardino Mountains in southern California. Federal Register.

[ref-66] Urban DL (2003).

[ref-67] Urban D, Keitt T (2001). Landscape connectivity: a graph-theoretic perspective. Ecology.

[ref-68] Vucetich JA, Nelson MP, Phillips MK (2006). The normative dimension and legal meaning of endangered and recovery in the US Endangered Species Act. Conservation Biology.

[ref-69] Wilcove DS, Rothstein D, Dubow J, Phillips A, Losos E (1998). Quantifying threats to imperiled species in the United States. Bioscience.

[ref-70] Williams DA, Wang YQ, Borchetta M, Gaines MS (2007). Genetic diversity and spatial structure of a keystone species in fragmented pine rockland habitat. Biological Conservation.

[ref-71] Wilson HB, Kendall BE, Possingham HP (2011). Variability in population abundance and the classification of extinction risk. Conservation Biology.

