# Supplementary figures and images for: Representing connectivity: quantifying effective habitat availability based on area and connectivity for conservation status assessment and recovery
Source: PeerJ. 2014 Oct 9;2:e622. doi: 10.7717/peerj.622 (PMC4194459; doi:10.7717/peerj.622)

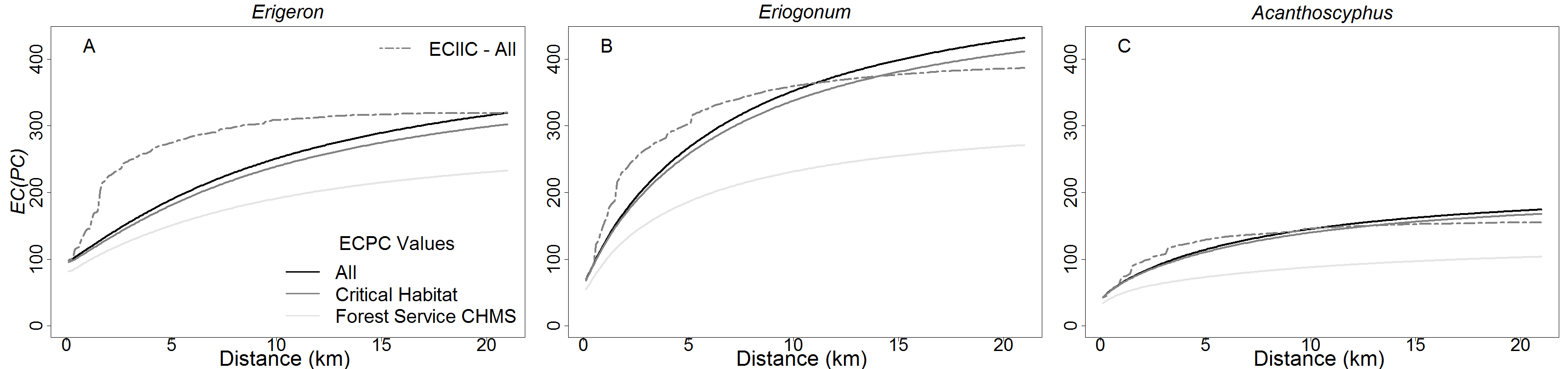

Supplement: Figure S1 — EC(PC) across a range of distance thresholds for all Erigeron parishii (A), Eriogonum ovalifolium var. vineum (B), and Acanthoscyphus parishii var. goodmaniana (C) patches in the full network, critical habitat, and the Forest Service’s carbonate habitat management strategy. PC was calculated with a 0.25 probability of dispersing at each selected threshold distance. EC(IIC) in the full landscape for each taxon is given for comparison. At the shortest distances EC(PC) was within 0.2–2% of EC(IIC) for full landscapes. EC(IIC) values then increase more rapidly at intermediate distances but maximum values of EC(PC) were ∼11% larger than EC(IIC) for Eriogonum and Acanthoscyphos, but differed by less than 1% for Erigeron. These relative values will vary with different probabilities of dispersal in PC. [file peerj-02-622-s001.png]
